# Antigenic structure of the human coronavirus OC43 spike reveals exposed and occluded neutralizing epitopes

**DOI:** 10.1038/s41467-022-30658-0

**Published:** 2022-05-25

**Authors:** Chunyan Wang, Emma L. Hesketh, Tatiana M. Shamorkina, Wentao Li, Peter J. Franken, Dubravka Drabek, Rien van Haperen, Sarah Townend, Frank J. M. van Kuppeveld, Frank Grosveld, Neil A. Ranson, Joost Snijder, Raoul J. de Groot, Daniel L. Hurdiss, Berend-Jan Bosch

**Affiliations:** 1 Virology Section, Infectious Diseases and Immunology Division, Department of Biomolecular Health Sciences, Faculty of Veterinary Medicine, Utrecht University, Utrecht, The Netherlands; 2 Astbury Centre Structural Molecular Biology, School Molecular and Cellular Biology, Faculty Biological Sciences, University of Leeds, Leeds, UK; 3 Biomolecular Mass Spectrometry & Proteomics, Bijvoet Center for Biomolecular Research, Department of Chemistry, Faculty of Science, Utrecht University, Utrecht, The Netherlands; 4 Department of Cell Biology, Erasmus Medical Center, Rotterdam, The Netherlands; 5 Harbour BioMed, Rotterdam, The Netherlands; 6 Present Address: State Key Laboratory of Agricultural Microbiology, College of Veterinary Medicine, Huazhong Agricultural University, Wuhan, P.R. China

**Keywords:** Viral immune evasion, Antibodies

## Abstract

Human coronavirus OC43 is a globally circulating common cold virus sustained by recurrent reinfections. How it persists in the population and defies existing herd immunity is unknown. Here we focus on viral glycoprotein S, the target for neutralizing antibodies, and provide an in-depth analysis of its antigenic structure. Neutralizing antibodies are directed to the sialoglycan-receptor binding site in S1_A_ domain, but, remarkably, also to S1_B_. The latter block infection yet do not prevent sialoglycan binding. While two distinct neutralizing S1_B_ epitopes are readily accessible in the prefusion S trimer, other sites are occluded such that their accessibility must be subject to conformational changes in S during cell-entry. While non-neutralizing antibodies were broadly reactive against a collection of natural OC43 variants, neutralizing antibodies generally displayed restricted binding breadth. Our data provide a structure-based understanding of protective immunity and adaptive evolution for this endemic coronavirus which emerged in humans long before SARS-CoV-2.

## Abstract (web summary)

Human coronavirus OC43 causes respiratory disease and is maintained in the human population through recurring infections. Here, by extensive structural analyses, the authors provide insights into the binding sites and breadth of neutralizing antibodies against this endemic coronavirus.

## Introduction

Coronaviruses, enveloped positive-strand RNA viruses of mammals and birds, pose a considerable zoonotic threat, as poignantly illustrated by the ongoing COVID-19 pandemic. Indeed, long before the emergence of severe acute respiratory syndrome coronavirus 2 (SARS-CoV-2) in 2019, four other CoVs arose from cross-species transmission to become established in the human population. NL63 and 229E are alphacoronaviruses in the subgenera Setraco- and Duvinacovirus, respectively. These viruses use bats as a natural host reservoir^1^, although the presumptive immediate ancestor of 229E is a virus natural to dromedary camels. HKU1 and OC43, like SARS-CoV-2, are in the genus Betacoronavirus but grouped in another subgenus called Embecovirus. Embecoviruses are found in large abundance in muroids and, from this natural reservoir, spilled into a variety of other mammals including livestock and companion animals. Whereas the origin of HKU1 is unknown, OC43 presumably arose from a relatively recent cross-species transmission of bovine coronavirus (BCoV). According to molecular clock analysis, the OC43-BCoV split was estimated to have occurred some 130 years ago^2–4^, whereas the most recent common ancestor of all extant OC43 variants was dated to the 1950’s^3–5^.

Collectively, the endemic human coronaviruses (HCoVs) account for an estimated 10–15% of acute respiratory diseases in both children and adults. Although generally associated with mild upper respiratory tract illness, they can also cause severe lower respiratory tract illness, including bronchitis and pneumonia, occasionally even with fatal outcomes. OC43 infections have also been associated with rare, neurological conditions^6–9^. As yet, there are no approved prophylactics or therapeutics for any of the four viruses.

The endemic HCoVs are maintained in the human population through continuous circulation, apparently sustained by frequent reinfections. Indeed, despite high seroprevalence levels already seen in children^10–12^, reinfections occur as soon as 6-8 months after the previous infection^13–15^. How HCoVs manage to do so is not known. Protective immunity is thought to be conferred predominantly by neutralizing antibodies directed against the spike (S) proteins, homotrimeric assemblies that mediate receptor-binding and membrane fusion during entry. Recent computational analyses revealed that specific regions of the 229E and OC43 S proteins are under strong positive selection indicative of adaptive evolution. In accordance, phylogenetic trees estimated for these S proteins have ladder-like topologies with long trunks and short terminal branches consistent with antigenic drift as a mechanism of immune escape^16^. In further support of this view, antisera raised against older strains of 229E are less effective in neutralizing contemporary field variants^17^. However, the epitopes and functional activities of neutralizing antibodies against the S proteins of the endemic human coronaviruses remain poorly defined. Understanding the molecular basis of viral neutralization by antibodies and the identification of key viral epitopes is essential to understand humoral immunity against the endemic HCoVs and to comprehend not only how they managed to colonize humans as a novel host but also how they escape herd immunity to persist in the population.

The human coronavirus OC43 is the most prevalent pathogen among the four endemic human coronaviruses causing a significant health burden worldwide. Its trimeric spike structure has recently been resolved in complex with its sialoside receptor^18,19^. Similar to other coronaviruses, the spike monomers are composed of two functional subunits—S1 and S2 that mediate binding to the host cell and fusion of the viral and cellular membranes, respectively. The N-terminal S1 subunit contains four core domains (S1_A_ through S1_D_) of which S1_A_ (also known as N-terminal domain or NTD) interacts with 9-*O*-acetylated sialic acids as the entry receptor^18,19^. While the S1_B_ (a.k.a. C-terminal domain or CTD) of other coronaviruses may be involved in binding to protein receptors, no function has been attributed to this domain for OC43^20–25^. Three copies of the S2 subunit, which contains the fusion peptide, can mediate viral fusion and cell entry. Despite the common presence and clinical significance of OC43, knowledge on the spike antigenic structure of this human pathogen is lacking.

In this work we studied the antigenic structure of the human coronavirus OC43 spike protein by characterizing the neutralizing capacity, binding sites and binding breadth of a panel of anti-OC43-S monoclonal antibodies using structural and functional approaches. We defined multiple vulnerable sites on the OC43 spike protein recognized by neutralizing monoclonal antibodies and showed that most neutralizing antibodies had limited binding breadth due to naturally occurring sequence diversity in the epitopes across OC43 isolates. Collectively, these results provide a structural basis for understanding humoral immunity and adaptive evolution for this endemic human coronavirus.

## Results

### Neutralizing human monoclonal antibodies targeting distinct antigenic regions on the OC43 S1 subunit

**Fig. 1:**
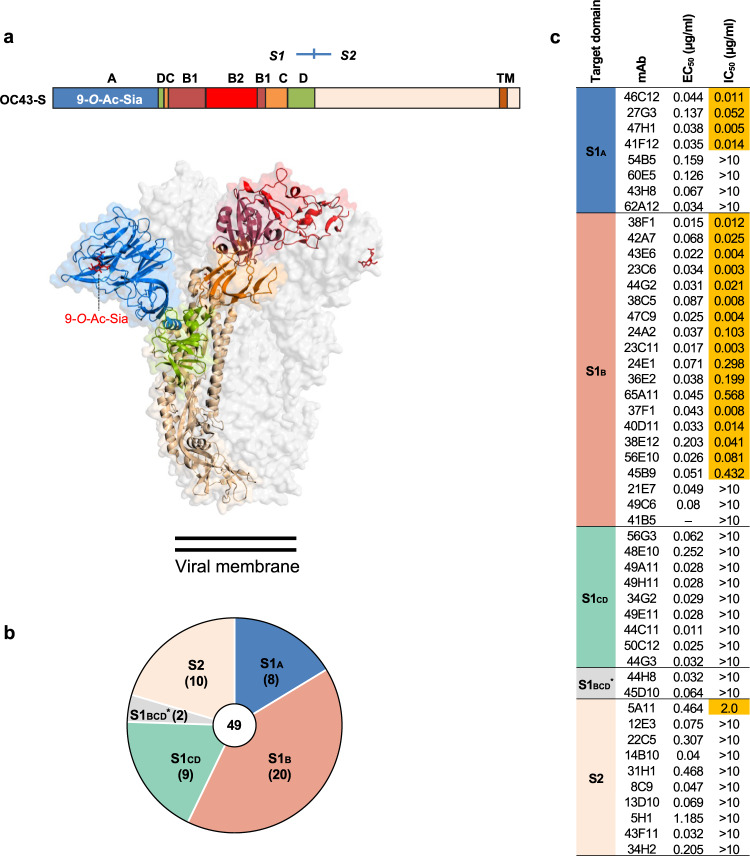
Binding properties and neutralization screening of OC43 S-specific H2L2 mAbs. **a** Organization of the OC43 spike (S) protein. Upper part: Linear diagram of OC43 S protein (drawn to scale). The S protein subunits (S1 and S2), S1 domains (A through D, with the B domain divided into the B1 and B2 subdomains), positions of the transmembrane domain (TM), and 9-*O*-acetylated sialic acid (9-*O*-Ac-Sia) receptor binding function are indicated. Lower part: cryo-EM structure of the trimeric OC43 S ectodomain (PDB: 6NZK) in transparent surface mode. The S protein trimer is composed of three S1 subunits that forms the membrane distal ‘head’ and three S2 subunits that constitutes the membrane proximal central ‘base’. Ribbon structure of one spike protomer is shown, with S domains colored as in schematic. The other two protomers are shown in light gray. The 9-*O*-Ac-Sia receptor is indicated in sticks and colored red. **b** Number of H2L2 mAbs that bind to the indicated OC43 S1 domains or S2, as determined by ELISA. S1_BCD_^*^: mAbs that are reactive to S1_BCD_ but not to the S1_B_ or S1_CD_. The total number of mAbs is shown in the center of the pie. **c** OC43 S-directed mAbs (49 in total, H2L2 format) were evaluated for ELISA-reactivity (shown as half-maximal effective concentrations (EC_50_)) against OC43 S_ecto_ (OC43 strain USA/1967) and neutralization potency (shown as half-maximal inhibitory concentrations (IC_50_)) against OC43 S VSV pseudovirus on HRT-18 cells. Antibodies are grouped by the targeted spike domains. Neutralizing antibodies are highlighted in orange. ‒, not tested. ELISA binding was performed twice independently. The EC_50_ and IC_50_ values from a representative experiment are shown, binding and neutralization curves are provided in Supplementary Fig. 1d and e.

**Fig. 2:**
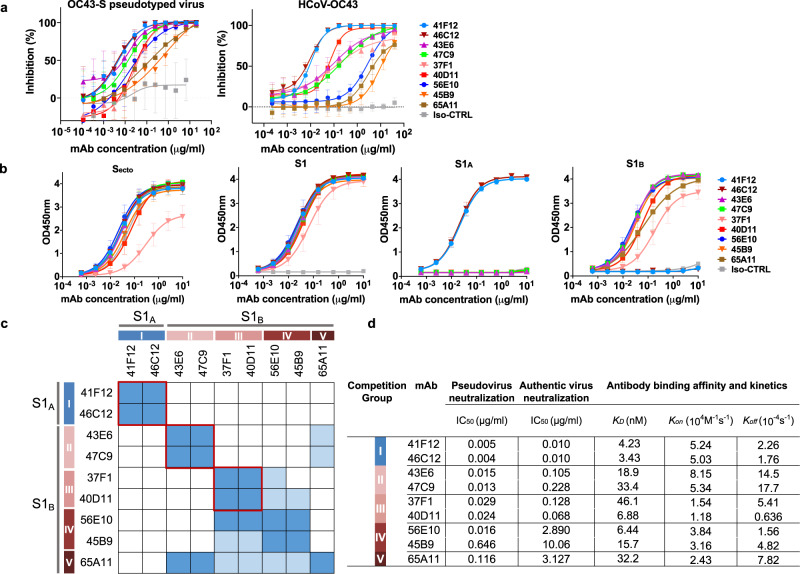
Neutralizing and binding profile of OC43 S1-reactive human mAbs. **a** Neutralizing activity of S1-directed human mAbs against OC43 S pseudotyped VSV (left panel) or authentic virus (USA/1967 ATCC strain, right panel) on HRT-18 cells. Data points represent means (±SD) of two independent experiments with six technical replicates. Iso-CTRL: antibody isotype control. **b** ELISA binding curves of mAbs to different types of OC43 S proteins. Data points represent means (±SD) of three independent experiments with each two technical replicates. Iso-CTRL: an anti-Strep-tag human monoclonal antibody was used as an antibody isotype control. **c** Heatmap showing binding competition of antibody pairs to the OC43 S1 protein, as determined by biolayer interferometry. Results are classified using color shading codes with a percentage of inhibition ≥75% in blue, <75% but ≥40% in light blue, and no shading for a percentage of inhibition <40%. Among the S1-binding antibodies, three mutually exclusive antibody groups (red rectangles, group I-III) were defined as well as two groups (IV and V) with a more miscellaneous binding competition profile. BLI sensorgrams showing the mAb binding competition profiles are shown in Supplementary Fig. 2. Each competition was performed twice independently, data from one representative experiment is shown. **d** Neutralizing potency and binding affinity of OC43 S1-directed human mAbs. Neutralization titers (IC_50_) were calculated based on inhibition curves shown in panel **a**. Antibody binding parameters determined by biolayer interferometry. BLI sensorgrams showing the kinetics of mAb binding to monomeric OC43 S1 antigen are shown in Supplementary Fig. 3.

To define neutralizing epitopes on the OC43 spike protein, we used an earlier established panel of hybridoma’s generated from spike-immunized H2L2 mice^26,27^. These transgenic H2L2 mice—encoding chimeric immunoglobulins with human variable heavy and light chains and murine constant regions—had been immunized with the trimeric spike ectodomains (S_ecto_) of three human-infecting betacoronaviruses OC43, SARS-CoV and MERS-CoV. The OC43 spike antigen used for mice immunization was from the prototype laboratory strain discovered in 1967 (USA/1967 strain) that was passaged several times in human embryonic tracheal organ culture, in suckling mouse brain, and in cell culture^3,28,29^, and which is the only strain that can be grown in tissue culture cells. We screened OC43 S_ecto_-reactive H2L2 monoclonal antibodies (mAbs)—using the hybridoma supernatants (75 in total), or upon mAb purification from cultured and cloned hybridoma’s (49 in total)—with respect to spike domain specificity, ELISA-based affinity and pseudovirus neutralizing activity, as well as the competitive binding profiles of S1-directed neutralizing antibodies by biolayer interferometry (BLI) (Fig. 1a–c, Supplementary Fig. 1a–f). Of the 49 H2L2 antibodies, 22 mAbs displayed neutralizing activity with epitopes in S1 (21 in total: 4 bound to S1_A_ and 17 to S1_B_ domain) and S2 (1 in total). Nine neutralizing antibodies directed against OC43 S1 were prioritized for in depth characterization and, prior to further analysis, reformatted to fully human IgG1 antibodies by cloning the genes of the variable region of light and heavy chains into mammalian expression vectors encoding the human constant regions. We evaluated these human antibodies for neutralization activity against VSV-based pseudovirus on HRT-18 cells (Fig. 2a). High neutralization potency was observed for mAbs 41F12 and 46C12 (IC_50_ values 0.005 and 0.004 μg/ml, respectively), followed by mAbs 43E6, 47C9, 37F1, 40D11 and 56E10 (IC_50_ values ranging between 0.013 and 0.029 μg/ml). Lower neutralizing potency was seen for 45B9 (IC_50_: 0.646 μg/ml) and 65A11 (IC_50_: 0.116 μg/ml). Neutralization assays performed with authentic OC43 virus (USA/1967 strain) yielded similar results (Fig. 2a). Two of the nine mAbs recognized epitopes in the S1_A_ domain (41F12 and 46C12) and the remaining seven in S1_B_ (43E6, 47C9, 37F1, 40D11, 56E10, 45B9, and 65A11) as demonstrated by ELISA using different OC43 spike antigen forms (Fig. 2b). To further characterize the antigenic sites on the OC43 S protein, we performed a BLI-based cross-competition binding assay with OC43 S1 protein-coated sensors (Fig. 2c, Supplementary Fig. 2). The levels of binding competition, ranging from maximal to non-detectable, allowed us to divide the S1 neutralizing antibodies into five distinct antibody competition groups, corroborating the competition groups observed for the H2L2 antibodies (Supplementary Fig. 1f). Group I antibodies included S1_A_-binding antibodies 41F12 and 46C12; group II to V comprise S1_B_-binding antibodies 43E6 and 47C9 (II), 37F1 and 40D11 (III), 56E10 and 45B9 (IV), and 65A11 (V). Group I, II, and III mAbs are non-competing with each other. Group IV and V mAbs have a more miscellaneous competition profile and show (partial) binding competition with mAbs from other groups. The binding kinetics of each of the nine mAbs were evaluated by BLI using OC43 S1 monomer as a substrate, revealing K_D_ values in the nanomolar range (3.4– 46.1 nM) (Fig. 2d, Supplementary Fig. 3).

### Neutralizing S1_A_-directed antibodies bind the sialoglycan-receptor site and preclude receptor engagement

**Fig. 3:**
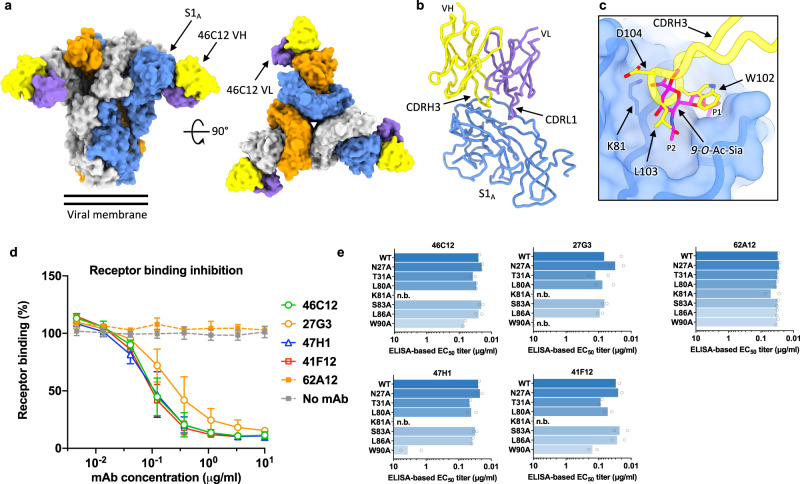
Neutralizing S1A binding antibodies recognize the sialoglycan-receptor binding site and block receptor binding. **a** Surface representation of the OC43 spike bound to three 46C12 antibody Fab fragments, shown as two orthogonal views. The threefold symmetry axis is denoted by a triangle. **b** Atomic model of a single OC43 S N-terminal domain in complex with 46C12 Fab fragment. **c** Zoomed-in view of the OC43 S 46C12 Fab binding site in transparent surface representation with the binding position of 9-*O*-Ac-Sia superposed (PDB: 6NZK) and the hydrophobic pockets P1 and P2 indicated. The CDRH3 loop of 46C12 would preclude sialic acid binding. In (**a**–**c**), each OC43 S protomer is colored distinctly (gray, blue, and orange), whereas the 46C12 heavy- and light-chain variable domains are colored yellow and purple, respectively. **d** Interference of monoclonal antibodies with receptor binding. OC43 S_ecto_ pre-incubated with serially diluted mAbs was added to ELISA plates coated with bovine submaxillary mucin (BSM) highly enriched in sialoglycan receptors^61^. Binding of OC43 S_ecto_ to BSM was detected using an HRP-conjugated antibody recognizing the C-terminal Strep-tag on OC43 S_ecto_. Data points represent means (±SD) of two independent experiments (*n* = 4). **e** ELISA-binding of S1_A_-reactive mAbs to S1_A_ proteins containing single amino acid substitution in 9-*O*-acetylated sialic acid receptor-interacting residues. The neutralizing S1_A_ mAbs 46C12, 27G3, 47H1, and 41F12 as well as the non-neutralizing S1_A_ mAb 62A12 were tested. Data are shown as mean EC_50_ values of two independent experiments with two technical replicates each. ELISA binding curves are provided in Supplementary Fig. 5. n.b., no binding.

To gain further insight into the location of the neutralizing epitopes present on the OC43 spike, we performed single-particle cryo-electron microscopy (cryo-EM) analysis (Supplementary Table 1, Supplementary Fig. 4). The cryo-EM structure of the OC43 spike ectodomain trimer complexed with the Fab fragment of the S1_A_-targeting antibody 46C12, at global resolution of 3.7 Å, revealed stochiometric binding (Fig. 3a, Supplementary Table 1). The Fab binds to the peripheral loops on the S1_A_ domain, with a total interface area of 908.5 Å^2^ (488.9 Å^2^ from the heavy chain and 419.6 Å^2^ from the light chain). The paratope comprises the CDRL1 and 2 loops, and the CDRH3 loop. The 46C12 epitope overlaps with the binding site for the sialoglycan receptor (Fig. 3a–c). Of note, the CDRH3 loop projects into the receptor-binding pocket on OC43 S, presumably preventing interaction of the sialoside receptor. The sidechain of L103, similarly to N-acetyl methyl of the receptor, projects into the P2 pocket of the receptor-binding site. The backbone amide of L103 forms a hydrogen bond with the carbonyl group of S1_A_ residue K81, similarly to the 5-nitrogen atom of 9-*O*-Ac-Sia. Moreover, K81 also forms a salt bridge with CDRH3 residue D104, reminiscent of the interaction formed by the 9-*O*-Ac-Me-Sia C1-carboxylate. Collectively, this demonstrates that 46C12 occludes the sialic acid-binding site through molecular mimicry.

We next evaluated the receptor binding interference by neutralizing S1_A_-binding antibodies in the ELISA-based spike-sialoglycan binding assay (Fig. 3d). The four neutralizing S1_A_-directed antibodies (46C12, 27G3, 47H1, and 41F12) all blocked binding of the trimeric S ectodomain (S_ecto_) to the receptor substrate in a concentration-dependent manner, whereas no binding interference was seen for the non-neutralizing S1_A_ antibody 62A12 (Fig. 3d). These data indicate that these neutralizing S1_A_-targeting antibodies bind sufficiently proximal to the sialoglycan-receptor binding site to compete with receptor binding, rationalizing their potent neutralizing activity. We subsequently tested antibody ELISA-reactivity to a panel of seven S1_A_ mutants with single-site alanine substitutions of residues known to be critical for sialoglycan-receptor binding^18,19^. Binding of all four neutralizing S1_A_ antibodies was abrogated by the K81A substitution, which for 46C12 is consistent with the notion that K81 in S1_A_ forms a salt bridge with D104 in the CDRH3 loop of 46C12 (Fig. 3c, e, Supplementary Fig. 5). In addition, binding of two neutralizing antibodies (47H1 and 27G3) was strongly reduced by the W90A mutation (Fig. 3e, Supplementary Fig. 5). The non-neutralizing S1_A_ antibody 62A12 retained binding to all mutants. Collectively, these data indicate that all S1_A_ antibodies with neutralizing activity bind a common antigenic site with modest differences in epitope fine specificity. They share functional activity and apparently prevent virus infection by blocking virus attachment to host cells through competitive binding to the sialoglycan-binding site.

### Neutralizing S1_B_-directed antibodies targeting non-overlapping, exposed epitopes on the trimeric spike

**Fig. 4:**
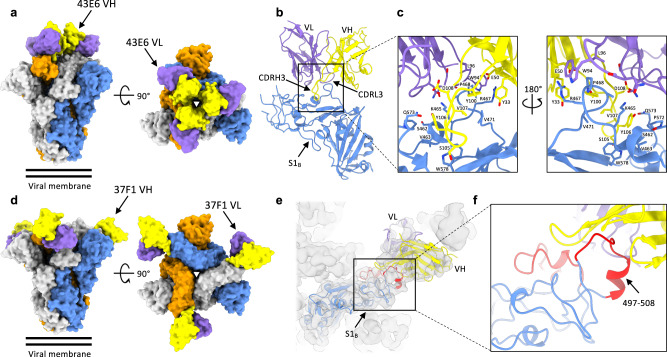
Distinct, non-overlapping neutralizing epitopes in S1B identified by cryo-electron microscopy. **a** Surface representation of the OC43 spike bound to three 43E6 antibody Fab fragments, shown as two orthogonal views. The threefold symmetry axis is denoted by a triangle. **b** Atomic model of a single OC43 S1_B_ domain in complex with the 43E6 Fab fragment. **c** Zoomed-in view of the OC43 S 43E6 Fab binding site. **d** Surface representation of the OC43 spike bound to three 37F1 antibody Fab fragments, shown as two orthogonal views. **e** Homology model of a single OC43 S1_B_ domain in complex with the 37F1 Fab fragment, fitted into the gaussian filtered EM density. The flexible loop regions that are not resolved in previous OC43 S structures are colored red. **f** Zoomed-in view of the OC43 S 37F1 Fab binding site. In (**a**–**f**), each OC43 S protomer is colored distinctly (gray, blue, and orange), whereas the heavy- and light-chain variable domains are colored yellow and purple, respectively.

We next determined cryo-EM structures of the OC43 spike in complex with Fab fragments of 43E6 and 47C9 antibodies (group II) at overall resolutions of 3.7 and 3.9 Å, respectively (Supplementary Table 1, Supplementary Figs. 6 and 7). These Fabs differ by only one residue in each of their heavy and light chain variable regions and, as expected, their binding modes are virtually identical, with the aligned Fab:S1_B_ complexes deviating by a root mean square deviation (RMSD) value of 0.22 Å across 265 Cα atoms. The higher-resolution structure of 43E6 was used for in-depth interaction analysis. Three copies of the Fab bind to S1_B_ on the most membrane distal region of the trimeric spike, close to the threefold symmetry axis, with a total interface area of 722.5 Å^2^ (488.5 Å^2^ from the heavy chain and 234 Å^2^ from the light chain) (Fig. 4a). The S1_B_ loop encompassing residues 473-483 is wedged between CDRL3 and CDRH3 of 43E6, participating in both hydrophilic and hydrophobic interactions (Fig. 4b). R467 projects towards the CDRH2 loop of 43E6, placing it in proximity to form a salt bridge with E50. The sidechain of CDRH3 residue Y106 extends into a shallow cleft formed by S1_B_ residues S462, V463, K465, P572 and Q573 (Fig. 4c).

We also determined the cryo-EM structure of the OC43 spike ectodomain in complex with the Fab fragment of the 37F1 (group III), albeit at a lower overall resolution of 4.4 Å (Supplementary Table 1, Supplementary Fig. 8). Nevertheless, the quality of the map was sufficient to dock a homology model of the Fab, revealing that 37F1 binds to an epitope at the tip of the S1_B_-B2 subdomain, distal to the spike 3-fold symmetry axis (Fig. 4d). Because the region of S1_B_ targeted by 37F1 is poorly resolved in previous OC43 spike structures^18^, we generated an S1_B_ homology model and docked this into our cryo-EM reconstruction (Fig. 4e and f). This indicated that the 37F1 epitope comprises a previously unmodeled loop, encompassing residues 497–508. Superposition with the 46C12 and 43E6 reveals that 37F1 binds a distinct and non-overlapping epitope, and approaches S1_B_ from a different angle than 43E6 (Supplementary Fig. 9).

No entry function has been attributed to S1_B_ so far. One possible explanation for their neutralizing activity is that S1_B_-directed antibodies sterically prevent receptor binding by domain A. However, preincubation of OC43 S trimers with the S1_B_-directed neutralizing mAbs did not block receptor interaction as evaluated in S_ecto_-sialoglycan binding assays (Supplementary Fig. 10a). In addition, as OC43 can hemagglutinate erythrocytes in sialoglycan-dependent manner^19,30^, we performed an hemagglutination inhibition (HAI) assay using the OC43 virus to assess interference of neutralizing S1_B_-directed antibodies with Spike-mediated sialoglycan interactions. In contrast to S1_A_-mAbs, none of the seven tested S1_B_-directed neutralizing mAbs exhibited HAI activity. Collectively, our data indicate that these antibodies neutralize infection by a mechanism that is independent of sialoglycan-receptor inhibition (Supplementary Fig. 10b).

### Neutralizing S1_B_-directed antibodies targeting cryptic epitopes in the closed S trimer

We next sought to structurally define the epitopes of the remaining S1_B_-directed neutralizing antibodies, including the competing pair of 56E10/45B9 and 65A11. However, single-particle cryo-EM reconstructions of spikes mixed with the Fabs of these antibodies only revealed the apo-form of the prefusion spike trimer. Moreover, BLI experiments showed that these S1_B_-directed antibodies, while binding to sensor-captured monomeric S1, were refractory to binding to the prefusion spike trimer (Supplementary Fig. 11). These data are in disparity with the ELISA data where binding activity by all mAbs was seen to the same prefusion spike trimer antigen coated on ELISA plates (Fig. 2b). We hypothesized that these antibodies target an epitope that is occluded in the prefusion spike trimer, and which may be exposed due to partial disassembly of S-ectodomains during adsorption to the ELISA plates.

**Fig. 5:**
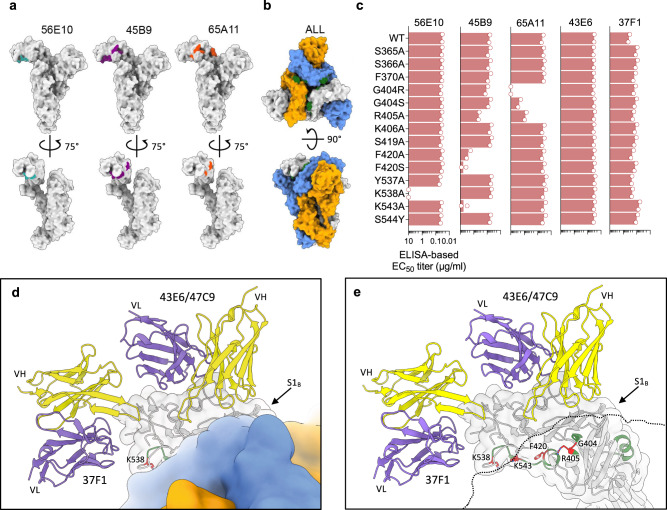
Cryptic neutralizing epitopes in S1B of the closed OC43 spike identified by HDX-MS. **a** Surface representation of the OC43 spike protomer in the context of the trimeric structure, shown in two orthogonal views (PDB: 6OHW), with the HDX-MS detected spike regions that are protected from deuteration by the 56E10, 45B9, and 65A11 Fabs. **b** Surface representation of the full OC43 S trimer: top view and side view (PDB: 6OHW). OC43 S protomers are colored in gray, blue, and orange. Epitopes for all three antibodies are shown together in the context of full OC43 spike and colored in forest green. **c** Impact of individual spike residue substitutions in HDX-MS identified epitope regions on mAb binding. Shown are ELISA-based EC_50_ binding titers of mAbs 56E10, 45B9, and 65A11, and two S1_B_ control mAbs 43E6 and 37F1 to S1 antigens containing individual single-site mutations. EC_50_ values were calculated from two independent experiments with four technical replicates shown. **d** Zoom-in view of the OC43 S trimer, centered on the S1_B_ domain and shown as a surface representation. One of the protomers is shown semi-transparent and overlaid with the ribbon diagram of S1_B_, 43E6, and 37F1. The HDX-MS identified residues are colored green and critical residues, confirmed by ELISA, are colored red and shown as sticks. **e** As shown in (**d**), but with two of the spike protomers shown as a silhouette to reveal the hidden epitope residues.

To identify the epitopes of 56E10, 45B9, and 65A11, we performed hydrogen-deuterium exchange mass spectrometry (HDX-MS) using monomeric S1 (Supplementary Figs. 12 and 13). We identified a total of 127 peptides suitable to monitor deuterium uptake in free S1 versus the Fab-bound complexes, covering 76% of the sequence overall, 81.3% of the S1_B_ domain (Supplementary Fig. 12). Protected regions were found for all three S1-Fab complexes, all mapping to the S1_B_ domain (Supplementary Fig. 13). Based on the deuterium uptake patterns found in overlapping peptides, the protected regions in the S1-45B9 complex were narrowed down to residues 399-406, 417-421, and 537-547. A single protected region was found in S1-56E10, corresponding also to residues 537-547, consistent with competition binding between 45B9 and 56E10. In the S1-65A11 complex, we found protection from deuteration in residues 361-371 and 399-406. All regions protected by 45B9, 56E10, and 65A11 map to regions of the S1_B_ domain that are indeed shielded in the structure of the closed OC43 spike trimer (Fig. 5a, b). The contribution of residues within the identified regions to antibody binding was assessed using S1 single substitution analysis. The S1_B_-reactive mAbs 43E6 (group II) and 37F1 (group III) were included as binding controls (Fig. 5c and Supplementary Fig. 14). F420S and K543A— and to a lesser extent F420A—abolished 45B9 binding indicating the importance of F420 and K543 to antibody recognition, further supported by their close proximity on the S1_B_ domain surface (Fig. 5d, e). Likewise, K538 was critical to binding by 56E10, as K538A eliminated 56E10 binding. Binding by 65A11 was lost by the G404R substitution, and impaired by the G404S and R405A mutations, indicating the importance of these consecutive spike residues for mAb binding. The mutational effects on mAb binding were antibody specific indicating distinct antibody binding modes. The positioning of the defined epitope residues for 56E10, 45B9, and 65A11 on the surface of the S1_B_ domain rationalizes the observed antibody binding competition profile in the BLI assay (Fig. 2c). The combined HDX-MS and mutagenesis data indicate that the opening of the S1 subunits, like observed in other betacoronavirus spikes, is required to allow exposure of the cryptic epitopes targeted by the neutralizing antibodies 56E10, 45B9, and 65A11.

### Viral escape pathways from neutralizing monoclonal antibodies

**Fig. 6:**
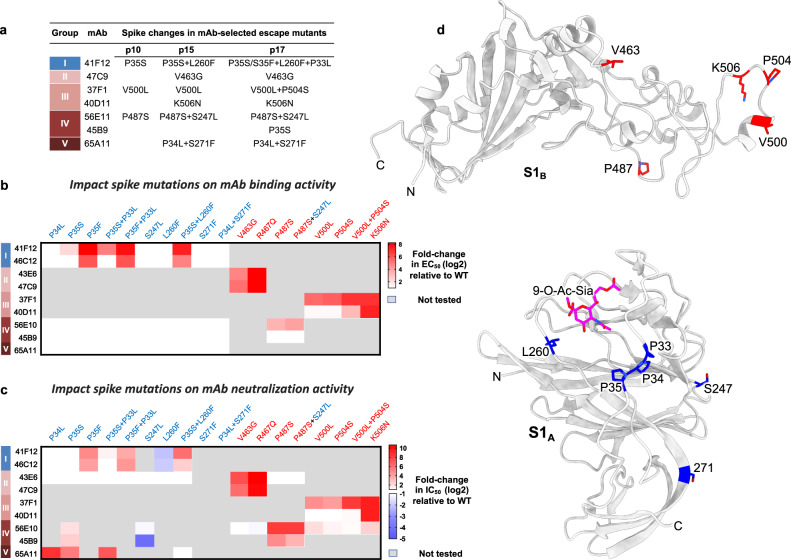
Effect of viral escape mutations on mAb binding and neutralization. **a** Spike mutations identified in OC43 following antibody selection. Antibody escape mutant viruses were generated by serial passaging of OC43 virus (strain USA/1967) on HRT-18 cells in the presence of each of the nine monoclonal antibodies. Spike genes of selected viruses were sequenced at different passages (passage 10, 15, and 17) using viral RNA extracted from cell supernatants. Spike mutations were identified in viruses following selection with mAbs 41F12, 47C9, 37F1, 40D11, 56E10, 45B9 and 65A11. **b**, **c** Effect of viral escape mutations on mAb binding and pseudovirus neutralization. **b** ELISA-based EC_50_ values of mAbs to S1 mutants were calculated and were visualized in a heatmap as (log2 transformed) EC_50_ titer shifts relative to wildtype control using GraphPad software (Prism 8.3.0). Mutations within S1_A_ and S1_B_ domains are colored in blue and red, respectively. Gray-colored boxes indicate combinations of mAbs and mutations that have not been tested. One independent experiment (two technical replicates) out of two is shown, and ELISA binding curves are provided in Supplementary Fig. 16. **c** Likewise, the impact of viral escape mutations on neutralization (log2 transformed IC_50_ shifts) using mutant OC43 S pseudoviruses was visualized. IC_50_ values represent means of ≥3 replicates, and neutralization curves are provided in Supplementary Fig. 17**. d** OC43 spike S1_B_ domain homology model (upper panel) and S1_A_ (lower panel) in cartoon representation with the antibody escape mutations highlighted.

To evaluate possible escape mechanisms of the neutralizing human mAbs, we serially passaged OC43 virus in the presence of each of the nine mAbs and sequenced the S gene of antibody escape mutant viruses. Under selective pressure of each of the neutralizing antibodies—except for 46C12 and 43E6—escape mutants readily emerged, encoding spike proteins with one or more amino acid substitutions in the S1 subunit (Fig. 6a, Supplementary Fig. 15). The impact of these mutations on antibody binding was evaluated by ELISA using a selection of S1 derivatives with each of the amino acid substitutions, either individually or in combination (Fig. 6b, Supplementary Fig. 16). In addition, mutations were tested for their ability to confer resistance to monoclonal antibodies using the VSV-based pseudovirus assay (Fig. 6c, Supplementary Fig. 17).

The S1_A_-targeting neutralizing 41F12 was selected for four amino acid changes in the S1_A_ domain (P35S, L260F, P35F, and P33L), all located near the rims of the sialic acid pocket. P35S was selected by a single nucleotide mutation (CCT to TCT) in two intermediate passages. The P35- > S- > F mutation requiring two nucleotide changes (CCT- > TCT- > TTT) occurred in the final passage. Whereas P35S slightly increased the EC_50_ titer for 41F12 by fivefold, the P35F mutation fully abrogated binding and conferred high resistance to neutralization (32-fold increment in IC_50_ titer), indicative for progressive adaptive evolution. The binding of 46C12 (group I) was reduced to a similar extent in accordance with its epitope overlapping with that of 41F12. The binding of control antibody 43E6, however with its epitope in S1_B_, was not affected by either of the mutations. L260F had no effect on binding by 41F12 and 46C12, but rather increased neutralization sensitivity by both antibodies. In combination with P35S, L260F causes a further decrease in binding and neutralization by both antibodies, suggesting epistatic interactions between the two residues.

The S1_B_-targeting neutralizing antibody 47C9 (group II) selected for a V463G change in the S1_B2_ subdomain. This mutation reduced binding and neutralization titers for 47C9 by 36- and 74-fold, respectively, with equivalent reductions seen for 43E6 (also group II), indicating its critical role for recognition by both antibodies.

The S1_B_-targeting mAbs 37F1 and 40D11 (group III) collectively selected for three single-site mutations located within a structurally unresolved seven-residue loop (^500^VGSGPGK^506^) at the tip of S1_B_ that is in the apparent vicinity of the bound 37F1 Fab (Fig. 4d–f). 37F1 selected for V500L and P504S, either of which significantly impaired 37F1 binding and strongly reduced 37F1-mediated neutralization, but not for 40D11. The combination of V500L and P504S conferred enhanced loss of binding and neutralization by 37F1, and marginal escape from binding and neutralization by 40D11. In contrast, 40D11 selected for K506N which conferred strong resistance to binding and neutralization both by 40D11 as well as by 37F1. These data confirm that both mAbs bind overlapping epitopes apparently with K506N critical to their binding.

Selection with S1_B_-directed mAbs 56E10 and 45B9 (group IV) yielded escape mutations in S1_A_ and S1_B_. The 56E10-selected P487S mutation conferred resistance to binding by 56E10, indicating that P487—as well as the closely positioned K538 (Fig. 5d)—contribute to 56E10 binding. Although P487S did not detectably reduce 45B9 binding, the mutation conferred resistance to neutralization to 56E10 as well as to 45B9. The 56E10-selected P487S mutant virus accrued another mutation upon further passaging in S1_A_ – S247L—which on its own imparted increased neutralization by both antibodies, without sensitizing their binding activity. Although located in different domains, P487S and S247L are closely positioned to each other in trimeric prefusion spike. However, the significance of S247L emergence under 56E10 antibody selection remains unclear as the combination of the 56E10-selected P487S and S247L mutations did not notably increase resistance phenotype relative to that of P487S alone. Selection with the S1_B_-directed 45B9 yielded the P35S mutation in the S1_A_ domain. As expected, P35S did not impair antibody binding to S1, but provided resistance to pseudovirus neutralization by 45B9, as well as by 56E10, implying an allosteric immune escape mechanism.

Following selection with the S1_B_-directed neutralizing antibody 65A11 (group V), two residue substitutions—P34L and S271F—were identified in S1_A_. In accordance with their position outside the epitope-containing domain, P34L and S271F did not impair 65A11 binding to S_ecto_. P34L however reduced neutralization of VSV-based pseudovirus to non-detectable levels. Generation of OC43 S pseudovirus carrying S271F or S271F + P34L was not successful, despite several attempts. In addition to P34L, the P35S mutation—selected by the S1_A_-specific antibody 41F12 and the S1_B_-directed 45B9—also reduced neutralization by 65A11.

Collectively, viral escape mutants were identified that conferred antibody escape either directly by reduction of the binding affinity of the antibody to its epitope or indirectly through a potential allosteric mechanism. Perhaps most surprisingly, P35S was identified as a common resistance mutation for antibodies that target non-overlapping epitopes in S1 separated by a considerable distance in the native S conformation. The P35S mutant and other mutations (P33L, P34L, and P35F) that emerged under antibody selection are part of a triple proline motif ‘^33^PPP^35^’ in USA/1967 located downstream of the L1 loop (^27^NDKDTG^32^)^18^ in S1_A_ that constitutes the rim of the receptor-binding site, and forms part of the structurally defined 46C12 epitope (Supplementary Fig. 19a and c). Whereas neutralization escape to S1_A_-directed 41F12 (group I) by P35S (and P35F) is rationalized by a diminished antibody binding affinity to its target, the occurrence of P35S in the OC43 S trimer distal to the S1_B_ residing epitopes of 56E10/45B9 (group IV) and 65A11 (group V) antibodies implicates an allosteric immune evasion mechanism, in which S1_A_-related conformational changes during entry may affect accessibility of neutralizing S1_B_ epitopes.

### S1 neutralizing antibodies are affected by genetic diversity in OC43 strains

**Fig. 7:**
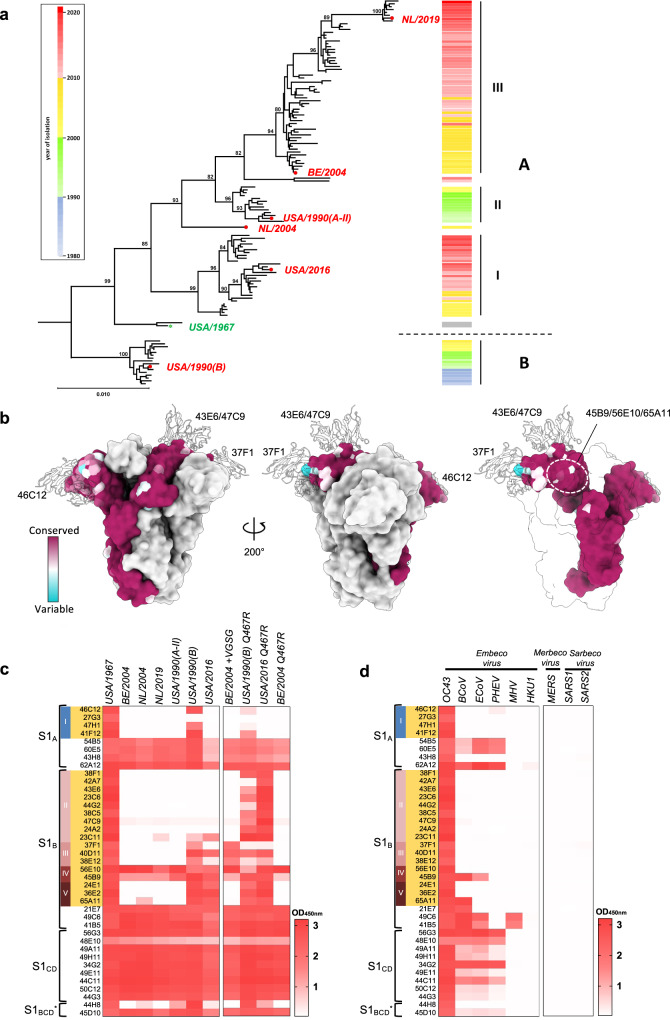
Binding breadth of OC43 S1-directed mAbs. **a** Evolution of OC43 S genes. Neighbor-Joining phylogeny constructed from an alignment of S genes of North-American/Eurasian field variants with bovine coronavirus as outgroup (see Supplementary Table 2). Branches were re-ordered from top to bottom based on collection date with the year of isolation indicated by color-coding in a sidebar (right) to visualize S evolution among and within distinct S lineages. Branchpoint confidence values >80 are given for all major nodes. The topological positions of S types selected for comparative analysis of antigenic properties are indicated by red dots; that of the oldest OC43 isolate, the USA/1967 strain, is indicated by a green star. Strain and GenBank identifiers of selected S types: NL/2019 (OK245434); BE/2004 (AY9034551); USA/1990(A-II) (KF530088); NL/2004 (OK245433); USA/2016 (MF314143.1); USA/1967 (AAT84354.1); USA/1990(B) (KF530065.1). **b** Spike protein sequence variability and position of neutralizing epitopes. One protomer in the OC43 spike trimer structure (PDB: 6OHW) is colored according to amino acid conservation across 228 OC43 S sequences. Ribbon diagrams of 46C12, 43E6 and 37F1 are overlaid and shown as a silhouette. In the right panel, two of the protomers are transparent and the shielded epitope region for 56E10, 45B9, and 65A11 are circled. GenBank identifiers of OC43 spike protein sequences are provided in Supplementary Fig. 18b. **c**, **d** Heatmap showing ELISA binding signal (OD450_nm_) of S1-directed mAbs (H2L2 format, 0.4 μg/ml) to (mutant) S1 proteins of OC43 isolates (**c**) or S1 proteins of multiple betacoronaviruses including human OC43, bovine BCoV, porcine PHEV and equine ECoV (all belonging to the Betacoronavirus-1 species within the Embecovirus subgenus), as well as the more distantly related embecoviruses MHV and HKU1, the merbecovirus MERS-CoV and two sarbecoviruses SARS-CoV and SARS-CoV-2 (**d**), with S1 of the orthologous USA/1967 as a reference. The antibody specificity (S1 domain and antibody competition group) is indicated on the left and neutralizing antibodies are highlighted in orange, similar as in Fig. 1c. Data from a representative experiment are shown.

Phylogenetic analysis to evaluate the diversification of US/Eurasian OC43 field variants and the evolution of the spike gene, in particular, suggested a split before 1967 into main groups A and B, and revealed the emergence and disappearance of major and minor S lineages over the last six decades (Fig. 7a, Supplementary Table 2). Plotting the naturally occurring S protein sequence diversity onto the spike structure showed that the variation coincided with the positions of the neutralizing epitopes of the structurally characterized Fabs (Fig. 7b, Supplementary Fig. 18b). The epitopes of 46C12 and 37F1 exhibit the greatest sequence variability (Fig. 7b). In contrast, the epitopes of the domain-occluded epitope regions contacted by 56E10, 45B9, and 65A11 exhibited a higher degree of conservation among OC43 isolates (Fig. 7b). Sequence alignments show that the sialoside receptor-interacting residues in the L1 and L2 loops are well conserved across OC43 spike proteins of naturally occurring OC43 strains (Supplementary Fig. 19a and c). However, considerable sequence variation is found in the periphery of these receptor-interacting residues in L1 and L2, as well as the binding site supporting L3 loop (^257^NSKLTL^262^) and include mutations that were selected by the S1_A_-directed mAb 41F12 (Supplementary Fig. 19a and c). Moreover, residues that are under positive selection also localize to the L1 to L3 loops that collectively compose the sialic acid binding site^18,31,32^. High level of sequence variation is also found in S1_B2_ subdomain containing the neutralizing epitopes of S1_B_-directed antibodies (Supplementary Fig. 19b, d and e). Particularly, the tip of B2 containing the 37F1 neutralizing epitope is characterized by three loops (K^494^-G^505^ loop, D^523^-L^525^ loop, and P^528^-T^536^ loop) that harbor significant levels of single-site amino acid mutations as well as two naturally occurring Indels, in K^494^-G^505^ loop (Indel3) and T^527^-T^536^ loop (Indel4) and four residues that are under positive selection (Supplementary Fig. 19b and d). In addition, a loop containing region (D^461^-D^476^) in B2 that composes a central part of the 47C9 mAb epitope—shows significant level of sequence variation as well as two residues that are under positive selection (Supplementary Fig. 19b and e).

To assess whether the naturally occurring sequence variation in OC43 S affected recognition by monoclonal antibodies, we screened the binding breadth of the 39 neutralizing and non-neutralizing S1-specific H2L2 mAbs displayed in Fig. 1b by ELISA using S1 antigens of six selected OC43 clinical isolates collected between 1990 and 2019 with spikes belonging to the distinct S lineages (Fig. 7a). To increase the throughput of the experiment, we tested ELISA binding at one concentration for each antibody. The heatmap showing the ELISA binding values (OD_450nm_) displays notable differences in the cross-reactivity profile for neutralizing and non-neutralizing antibodies (Fig. 7c). Cross-binding capability towards S1 of OC43 strains was dominant among non-neutralizing antibodies. All 18 non-neutralizing antibodies—with sole exception of 44H8—were broadly reactive, recognizing the S1 antigens of each of the six selected clinical isolates. In contrast, among the 21 neutralizing S1 antibodies, only 56E10 and 45B9—both targeting an occluded epitope—recognized all six strains. Cross-reactivity for the other neutralizing mAbs (group I, II, III, and V) was largely restricted to the USA/1990(B) and USA/2016 strains that are representatives of S lineages B and A-I, and virtually absent against S1 antigens from the other selected strains.

By evaluating the natural variation in the epitope regions, we could rationalize cross-binding diversity and further validate epitope positions for a number of antibodies. Relative to the orthologous USA/1967 spike, we observed an R467Q substitution in the 43E6 epitope in all six selected clinical isolates which may explain resistance to binding by this and other group II mAbs, as it prevents formation of a stabilizing salt bridge with the heavy chain of 43E6 (Fig. 4c, Supplementary Fig. 19b). Indeed, the reciprocal Q467R substitution in S1 proteins of USA/1990(B) and USA/2016 viruses resulted in gain of binding by most group II mAbs (Fig. 7c), indicating that the lack of antibody cross-reactivity resulted from a single residue change. Gain of binding by group II mAbs was not seen for S1 of the BE/2004 isolate upon Q467R substitution, indicating that additional epitope sequence diversity in S1 of this strain accounts for the lack of antibody reactivity.

Some of the naturally occurring mutations correspond with the identified viral mutations that confer escape from the neutralizing antibodies including P35S, P35F, L260F, and P504S, further supporting viral antigenic evolution (Supplementary Fig. 19a and b). In addition to the single-site amino acid mutations, insertions and deletions (Indels) in the epitope loops may contribute to antigenic diversity. OC43 spike sequences contain multiple regions with Indels which are exclusively found in the S1 subunit (Supplementary Fig. 19a and b)^33^. One of the Indels (Indel3) is located in the K^494^-G^505^ loop in the S1_B_ region contacted by the group III mAb 37F1. A four-residue deletion (^500^VGSG^503^) in this region differentiates the tested S1 antigens of strains reactive to group III mAbs from those that lack binding, including the BE/2004 strain. Insertion of this motif in S1 of the BE/2004 strain resulted in a gain of binding by group III antibodies (Fig. 7c).

These data suggest antigenic differences in neutralizing epitopes in the spike protein across OC43 strains and support the notion of selective evolutionary pressure on antigenic sites in OC43 S recognized by neutralizing antibodies that is compatible with antigenic drift.

### Binding breadth of S1 antibodies across betacoronaviruses

To determine cross reactivity beyond OC43, we screened the S1 mAbs in ELISA-binding assays using S1 antigens from multiple betacoronaviruses, including those of other members of the species Betacoronavirus-1, i.e., the presumptive OC43 ancestor bovine coronavirus (BCoV), porcine hemagglutinating encephalomyelitis virus (PHEV) and equive coronavirus (ECoV), as well as those of more distantly related embecoviruses, mouse hepatitis virus (MHV) and HCoV HKU1, merbecovirus MERS-CoV and sarbecoviruses SARS-CoV and SARS-CoV-2. Out of all mAbs, only 2—45B9 (group IV) and 65A11 (group V)—cross-reacted with BCoV S1, with 45B9 showing additional binding to S1 of ECoV (Fig. 7d). Consistent with their cross-binding reactivity, both antibodies neutralized BCoV S pseudotyped virus (Supplementary Fig. 20). In contrast to the mAbs, most of the 18 non-neutralizing S1-antibodies showed broad binding capacity against S1 antigens of other members of the Betacoronavirus-1 species (BCoV, ECoV and PHEV): 16 mAbs recognized BCoV S1, 15 mAbs recognized both BCoV and ECoV S1, and 13 mAbs recognized all three S1s (Fig. 7d). Two non-neutralizing S1 mAbs (49C6 and 41B5) also bound to MHV S1 and a single one, 62A12, showed modest cross reactivity towards HKU1 S1. None of them detectably bound to S1 of MERS-CoV, SARS-CoV or SARS-CoV-2. The findings show that the neutralizing epitopes in S1 of the OC43/USA/1967 prototype strain are poorly conserved even in its closest relatives within the Betacoronavirus-1 species. Conversely, most of the non-neutralizing epitopes were conserved at the species level, but, with few exceptions, not in members of other embecovirus species, and not in all members from other Betacoronavirus subgenera. Thus, serological cross-reactivity across embecoviruses at the level of the S protein may be largely based on a limited number of subgenus-wide conserved non-neutralizing epitopes.

## Discussion

We characterized a panel of monoclonal antibodies against the HCoV OC43 spike protein. Antigenic mapping of the neutralizing antibodies revealed multiple sites of vulnerability on the spike protein of this endemic coronavirus. The combined structural and in vitro data demonstrated the presence of three non-overlapping neutralizing epitopes on the surface of the prefusion S trimer, represented by the 46C12, 43E6, and 37F1 epitopes. The S1_A_-directed neutralizing antibodies recognize the S1_A_ receptor binding site for 9-*O*-Ac-Sia and directly block virus binding to sialoglycans by preventing virion-ligand interaction. These findings are in line with previous observations that attachment to sialoglycans is critical for OC43 infection. Surprisingly, however, most neutralizing mAbs were directed not against S1_A_ but against the S1_B_ domain, and, whereas in S1_A_ we identified only a single neutralizing epitope, four distinct epitopes were found in S1_B_, two of which surface-exposed in the closed prefusion S trimer conformation, the others hidden. The S1_B_-directed antibodies neutralize OC43 infection via an unknown mechanism, independent of sialoglycan receptor binding inhibition, because none of them prevented virion attachment to 9-*O*-Ac-Sia. The presence of multiple, non-overlapping sites of vulnerability in S1_B_ suggests a crucial role of this domain in entry not appreciated before. Such a role may involve the inhibition of spike conformational changes that lead to entry, or, alternatively, interactions with a putative, second receptor. This latter scenario has been proposed for the related HCoV HKU1, based on the observation that preincubation of cells with purified S1_B_ blocks HKU1 cell entry and that mAbs targeting the tip of this domain neutralize infection^34,35^.

Intriguingly, we found that three neutralizing antibodies—56E10, 45B9 and 65A11—bind cryptic epitopes that are buried in the closed, prefusion S trimer. Epitopes occluded in the closed spike trimer state have been identified for SARS-CoV-2 which only can be bound by neutralizing antibodies when the receptor-binding domain is in the up conformation^36^. Alternative spike conformations for OC43, however, have not been captured thus far by cryo-EM^18,37^. The occluded binding sites of these neutralizing antibodies nevertheless can be taken as an indication for conformational flexibility of the OC43 spike protein (e.g. during virus entry) which leads to the exposure of the cryptic neutralizing epitopes in S1_B_. The masked nature of the epitopes is further supported by the identification of mutations in the S1_A_ receptor binding domain that provide neutralization resistance to all three S1_B_-directed 56E10, 45B9, and 65A11, suggesting that the antibody escape mechanism may be allosteric and conceivably related to receptor binding induced conformational changes.

Using the library of mAbs raised against the OC43/1967 spike protein, we observed a strong conservation of non-neutralizing epitopes in the spike’s S1 subunit across OC43 strains as well as related embecoviruses. In contrast to non-neutralizing antibodies, the neutralizing antibodies tend to have restricted binding specificity indicating antigenic variability in neutralizing epitopes on spike proteins. The structurally defined, accessible neutralizing epitopes on the prefusion OC43 spike trimer strikingly map to regions of high sequence variability across OC43 isolates, indicating that these antigenic sites of OC43 are tolerant of amino acid substitutions and frequently occurring insertions/deletions that restrict antibody binding breadth, which is a commonly found evolutionary signature in viral glycoproteins under immune selection^38,39^. Much of the antigenic variation in the OC43 spike protein residues in the receptor-binding area. While the sialoglycan receptor-interacting residues are functionally conserved, we observed significant sequence variation in other regions of the receptor-binding motif across OC43 isolates, including the identified antibody escape mutations that conferred neutralization resistance. Similar observations have been made for influenza virus^38^, as well as for some coronaviruses that interact with protein receptors SARS-CoV-2 and 229E^40–42^. Apart from the strain diversity in the receptor-binding motif, a significant part of sequence variation is concentrated in the B2 subdomain of S1_B_, which overlaps with the neutralizing epitopes of group II and III mAbs rationalizing the observed diversity in binding breadth of the isolated neutralizing antibodies. Extended binding breadth was observed in particular for antibodies that target occluded epitopes, consistent with low sequence variability in their cognate binding site in S1_B_ among circulating OC43 variants. As highlighted by 45B9, such antibodies may even cross-neutralize related betacoronaviruses to provide cross-protection.

Collectively, we structurally defined neutralizing epitopes on the surface of OC43 spike trimer and demonstrated that these regions exhibit antigenic variability across OC43 isolates. Even small changes in the key neutralizing epitopes of antigenically variable pathogens may allow significant viral escape from immunity induced by prior infection or vaccination, which is illustrated by the need for annual assessment of influenza virus vaccine efficacy^41,43^. Recent computational studies on spike sequences of OC43 natural isolates collected since 1967 indicate substantial adaptive evolution, particularly in the S1 subunit^16,31^. Residues defined to be under purifying selection localized to the three distinct solvent-exposed neutralizing epitopes in the S1_A_ and S1_B_ domains identified in this study, providing definitive evidence that the OC43 spike protein is a target of immunological pressure in humans.

Phylogenetic analysis suggests that at any time during the last six decades OC43 variants with antigenically distinct S proteins co-circulated. A split to have occurred before 1967 gave rise to two major OC43 clades with S types provisionally designated A and B. The type A subsequently diverged into several co-evolving lineages, the most populous ones tentatively designated A-I through A-III (Fig. 7a), with progressive intra-lineage accumulation of mutations in neutralization epitopes. Intriguingly, clade B and lineage A-II S types seem to have gone extinct in the early 2000s and to have been displaced largely by A-I- and A-III-type spikes, which emerged around that same time and representatives of which still dominate the field to date as based on the frequency of isolation and widespread geographic occurrence. Komabayashi et al.^44^ from their longitudinal study on the occurrence of OC43 between 2010 and 2017 in Japan noted that viruses from different genotypes but with either A-I or A-III type S proteins (corresponding to their groups B and D, respectively) seemed to yearly alternate in the population. The combined observations of us and others indeed would support a model for the continued persistence of OC43 in the human population facilitated by alternating re-infections with antigenically distinct co-circulating OC43 variants and short-lived or incomplete humoral immunity in the population. More research is needed to monitor genetic diversity and evolutionary dynamics of OC43 and other endemic human coronaviruses, and to study the impact of antigenic changes in evading host immunity and in driving viral epidemics. The neutralizing epitopes on OC43 S, identified here, provide a structural framework to understand the adaptive evolution of the endemic OC43 coronavirus in response to humoral immunity, and are instructive for the development of pharmaceutical interventions. Moreover, our findings may be predictive of the long-term antigenic evolution of SARS-CoV-2 which emerged in humans only since late 2019 but that is projected to become endemic.

## Methods

### Viruses and cells

HRT-18 cells (ATCC® CCL-244™) were maintained in DMEM containing 10% fetal calf serum (FCS), penicillin (100 IU/mL), and streptomycin (100 μg/mL). The prototypic OC43 strain USA/1967 (VR-759) purchased from the American Type Culture Collection (ATCC), were propagated in HRT-18 cells.

### Expression and purification of coronavirus spike proteins

Gene fragments encoding the human coronavirus OC43 spike ectodomain (spike residues 15–1263) of the prototype OC43 strain USA/1967 (ATCC strain VR-759: GenBank: AAT84354.1) and S2 ectodomain (amino acid 759-1263) were amplified by RT-PCR from viral genome and cloned in-frame between a CD5 N-terminal signal peptide (MPMGSLQPLATLYLLGMLVASVLA) and a GCN4 trimerization motif (IKRMKQIEDKIEEIESKQKKIENEIARIKKIK) followed by a Strep-tag (WSHPQFEK) purification tag in the modified pCAGGS mammalian expression vector. The furin cleavage site *R*^754^*R*S*R*G^758^ at S1/S2 junction was mutated to *GG*S*G*G, to prevent cleavage by furin at this position as described previously^18^. Recombinant OC43 S ectodomain and S2 ectodomain were transiently expressed in HEK-293T cells and purified from culture supernatant using streptactin beads (IBA) following the manufacturer’s protocol. The genes encoding OC43 S1 subunit (amino acid 1-752), S1_A_ (amino acid 1-306), S1_BCD_ (amino acid 303-753), S1_B_ (amino acid 331-602) and S1_CD_ (amino acid 303-330-Gly Ser linker-611-753) were cloned in-frame in pCAGGS mammalian expression vector C-terminally extended with a thrombin cleavage site followed by the Fc region of human IgG, and were transiently expressed in HEK-293T cells and purified from culture supernatant using Protein A sepharose (IBA) according to the manufacturer’s instruction. All recombinant OC43 variants were generated using the same strategy. Genes encoding the S1 subunit of the OC43 isolates (amino acid 1-759, strain: NL/2004, GenBank: OK245433 and amino acid 1-758, strain: NL/2019, GenBank: OK245434) were amplified by RT-PCR from viral genome, genes encoding S1 of the OC43 isolates (BE/2004; GenBank: AAX84792.1; USA/1990(B); GenBank: AGT51461.1; USA/1990(A-II); GenBank: AGT51690.1, USA/2016; GenBank: AVQ05264.1) were synthesized by GenScript USA Inc. and the sequences were codon-optimized to maximize expression in the HEK-293T expression system. Expression of S1 subunits of spike proteins of BCoV (amino acid 1-763, strain Mebus, UniProtKB: P15777.1), ECoV (amino acid 1-762, strain NC99, GenBank: EF446615.1), PHEV (amino acid 1-750, strain UU, GenBank: ASB17086.1), MHV (amino acid 1-711, strain A59, UniProtKB: P11224.2), HKU1 (amino acid 1-750, strain Caen1, GenBank: ADN03339.1), MERS-CoV (amino acid 1-747, strain EMC, GenBank: YP_009047204.1), SARS-CoV (amino acid 1-676, strain WH20, GenBank: AAX16192.1) and SARS-CoV-2 (amino acid 1-682, strain Wuhan-Hu-1, GenBank: QHD43416.1) was performed as described previously^27,45^. Briefly, S1 genes were cloned into the pCAGGS expression vector in frame with the human Fc region and produced from HEK-293T cells as described above.

### Hybridoma culturing and purification of anti-OC43 S H2L2 mAbs

Hybridomas from spike-immunized H2L2 mice had been generated earlier^26,27^. Hybridoma’s were cultured in serum- and protein-free medium for hybridoma culturing (PFHM-II (1X), Gibco) with addition of non-essential amino acids (100X NEAA, Biowhittaker Lonza, cat. no. BE13-114E). H2L2 antibodies were purified from hybridoma culture supernatants using Protein-G affinity chromatography. Purified antibodies were stored at 4 °C for further use.

### Production of recombinant human monoclonal antibodies

Production of recombinant human antibodies using HEK-293T was described previously^46^. Briefly, the variable heavy (VH) and light (VL) chain sequences were amplified from cDNA and separately cloned into the expression plasmids with human IgG1 heavy chain and human kappa chain constant regions, respectively (InvivoGen). Both plasmids contain the interleukin-2 signal sequence to enable the efficient secretion of recombinant antibodies. Recombinant human antibodies were expressed in HEK-293T cells following transient transfection with pairs of the IgG1 heavy and light chain expression plasmids according to protocols from InvivoGen. Recombinant antibodies were purified using Protein A Sepharose (IBA) according to the manufacturer’s instructions.

### Antibody binding to coronavirus spike antigens analyzed by ELISA

ELISA analysis of antibody binding was performed as described previously with minor changes^26^. Purified antigens were coated onto 96-well NUNC Maxisorp plates (Thermo Scientific) at room temperature (RT) for 3 h followed by three washing steps with Phosphate Saline Buffer (PBS) containing 0.05% Tween-20. Plates were blocked with 5% milk (Protifar, Nutricia) in PBS with 0.1% Tween-20 at 4 °C overnight. Antibodies were allowed to bind to the plates at fourfold serial dilutions, starting at 10 μg/ml diluted in PBS containing 3% BSA and 0.1% Tween20, at RT for 1 h. Antibody binding to the spike proteins was determined using a 1:2000 diluted HRP-conjugated goat anti-human IgG (for human mAbs, ITK Southern Biotech, cat. no. #2040-05) or mouse anti-rat IgG1/2b/2c (for H2L2 mAbs, Absea Biotechnology Ltd., cat. no. KT96/KT98/KT99) for 1 h at RT and tetramethylbenzidine substrate (BioFX). Readout for binding was done at 450 nm (OD_450_) using the ELISA plate reader (EL-808, Biotek). Half-maximum effective concentration (EC_50_) binding values were calculated by 4-parameter logistic regression on the binding curves using GraphPad Prism version 8.3.0.

### Antibody binding kinetics and affinity measurement using biolayer interferometry

The measurement of binding kinetics and affinity of antibodies to monomeric OC43 S1 was performed using biolayer interferometry (Octet RED384 machine) as described before^26,27^. Briefly, fully human antibodies with optimal concentration (44 nM) which showed the desired loading curve characteristics and high signal in the association step were loaded onto Protein A biosensors for 10 min. The binding of OC43 S1 was performed by incubating the biosensor with various concentrations of recombinant OC43 S1 protein (400-200-100-50 nM) for 10 min followed by a long dissociation step (60 min) to observe the decrease of the binding response. The affinity constant K_D_ was calculated using 1:1 Langmuir binding model on Fortebio Data Analysis 7.0 software.

### Pseudovirus neutralization assay

The production of the OC43 S pseudotyped vesicular stomatitis virus (VSV) and the pseudovirus neutralization assay were performed as described previously^18,19,26,27^. In brief, HEK-293T cells at 70~80% confluency were transfected with the pCAGGS expression vectors encoding full-length OC43 S and BCoV S with a C-terminal cytoplasmic tail 17-aa truncation to increase cell surface expression levels. Cells were co-transfected with a pCAGGS vector encoding the Fc-tagged bovine coronavirus hemagglutinin esterase (HE-Fc) protein at molar ratios of 8:1 (S:HE-Fc). Forty-eight hours post transfection, cells were infected with the VSV-G pseudotyped VSVΔG bearing the firefly (*Photinus pyralis*) luciferase reporter gene at a MOI of 1. Twenty-four hours later, supernatant was harvested and filtered through 0.45 μm membrane. Pseudotyped VSV virus in the supernatant was titrated on monolayer HRT-18 cells. In the virus neutralization assay, threefold serially diluted mAbs starting from 40 μg/ml were pre-incubated with an equal volume of virus at RT for 1 h, and then inoculated on HRT-18 cells, and further incubated at 37 °C. After 20 h, cells were washed once with PBS and lysed with cell lysis buffer (Promega). The expression of firefly luciferase was measured on a Berthold Centro LB 960 plate luminometer using D-luciferin as a substrate (Promega). The percentage of infection was calculated as the ratio of luciferase readout in the presence of mAbs normalized to luciferase readout in the absence of mAb. The half maximal inhibitory concentrations (IC_50_) were determined using 4-parameter logistic regression (GraphPad Prism v8.3.0).

### Live virus neutralization assay

Monoclonal antibodies were threefold serially diluted and mixed with 100 TCID_50_ of OC43 (strain USA/1967) for 1 hour at RT. Antibody-virus mixtures were subsequently added to HRT-18 cells. Cells were washed, fixed, and permeabilized 12 h post infection. OC43-infected cells were detected by immunofluorescence. Briefly, cells were fixed by incubation with 2% paraformaldehyde in PBS for 20 min at RT before 0.1% Triton-100 permeabilization. Cells were subsequently incubated with a mixture of two mAbs 56E10 and 41F12 (both at 0.5 μg/ml) at RT, followed by incubation with 1:200 diluted Alexa Fluor 488 conjugated goat anti-human IgG antibody (Invitrogen, Thermo Fisher Scientific, cat. no. A-11013) for 45 min at RT. The plates were scanned on the Amersham Typhoon Biomolecular Imager (channel Cy2; resolution 10 mm; GE Healthcare). Infected cells were quantified using ImageQuantTL 8.2 image analysis software (GE Healthcare). The half-maximal inhibitory concentrations (IC_50_) were determined using 4-parameter logistic regression (GraphPad Prism version 8.3.0).

### Receptor binding inhibition assay

The receptor binding inhibition assay was performed as described previously with minor changes^26,27^. The bovine submaxillary mucin (BSM, Sigma-Aldrich) was coated on NUNC Maxisorp plates (Thermo Scientific) at 100 ng/well at RT for 3 h. Plates were washed three times with PBS containing 0.05% Tween-20 and blocked with 5% milk (Protifar, Nutricia) in PBS containing 0.1% Tween-20 at 4 °C overnight. Recombinant OC43 S ectodomain and serially diluted mAbs were mixed and incubated for 1 h at RT. The mixture was added to the plate for 1 h at RT, after which plates were washed three times. Binding of OC43 S ectodomain to BSM was detected using 1:2000 diluted HRP-conjugated anti-StrepMAb (IBA) that recognizes the Strep-tag affinity tag on the OC43 S ectodomain. Detection of HRP activity was performed as described above (ELISA section).

### Hemagglutination inhibition assay

The classical hemagglutination inhibition assay was performed according to standard procedures. Briefly, threefold dilutions of monoclonal antibodies were preincubated with an equal volume of 4 hemagglutinating units of OC43 (ATCC strain) for 1 h at room temperature (final volume 50 μl), and the mixture was subsequently mixed with 50 μl of a rat erythrocyte suspension (*Rattus norvegicus* strain Wistar; 0.5% in PBS) in V-bottom, 96-well plates (Greiner Bio-One). Plates were incubated overnight at 4 °C, after which the hemagglutination inhibition was assessed.

### Antibody binding competition assay

Antibody binding competition was performed using biolayer interferometry (Octet Red; ForteBio) as described previously with minor changes^26,46^. In brief, 50 μg/ml of human Fc-tagged OC43 S1 was immobilized onto protein A biosensor. After a brief washing step, the sensors were saturated with rabbit IgG (50 μg/ml, Dako) for 30 min. The biosensor tips were then immersed into a well containing primary mAb (mAb 1) at a concentration of 50 μg/ml for 15 min to allow saturation of binding to the immobilized antigen and subsequently into a well containing the competing mAb (mAb 2) at a concentration of 50 μg/ml for 15 min. A 5-min washing step in PBS was included in between steps. Binding of mAb 2 to OC43 S1 protein in the presence of saturating concentration of mAb 1 was measured and normalized to binding in the presence of an irrelevant anti-MERS S2 mAb 1.6C7. ≥ 75% inhibition: full competition, <75% but ≥40% inhibition: partial competition, and <40% inhibition: no competition^47^.

### Generation and analysis of neutralization escape mutants

A fivefold serially diluted range of each respective mAb was pre-incubated with an equal volume of OC43 virus (strain USA/1967) at RT for 1 h, and then inoculated on HRT-18 cells, and further incubated at 37 °C. Supernatants containing progeny virus were harvested at 72 h post infection and infection of cells was visualized by immunofluorescence, as described above. The virus infection indicated by the fluorescence was tested under the EVOS FL fluorescence microscope (Thermo Fisher Scientific, the Netherlands). Subsequent passages in the presence of antibodies were established using virus-containing supernatant from the passage with the highest concentration of antibody that permitted minimally ±30% infection of cells. This procedure was continued up to p17. Viral RNA from 500 μl aliquots of viruses from three passages (passages 10, 15, and 17) were isolated using the NucleoSpin RNA Virus kit (Macherey-Nagel), followed by conventional RT-PCR and the S genes of individual antibody-treated virus were sequenced by Sanger sequencing. The effect of emerging escape mutations on antibody binding and neutralization was tested by ELISA and pseudotyped virus, respectively, as described above.

### Preparation of Fab fragment from IgG

All Fabs were digested from IgG with papain using a Pierce Fab Preparation Kit (Thermo Fisher Scientific), following the manufacturer’s standard protocol.

### Cryo-EM grid preparation

Purified OC43 spike ectodomains (1 mg/ml) and antibody Fab fragments were incubated individually for 5 min at approximately a 1:1 molar ratio of spike monomer to Fab fragment prior to loading onto an EM grid. Quantifoil R2/2 grids were glow-discharged for 30 s prior to application of 3 µl of virus-fab mixture. Residual liquid was removed from grids using an FEI vitrobot (mark IV, ThermoFisher Scientific), grids were immediately vitrified by plunge freezing in liquid nitrogen-cooled liquid ethane.

### Cryo-EM data acquisition

Cryo-EM data were collected on a Titan Krios (ThermoFisher Scientific) operating at 300 kV with a Falcon III direct detector (ThermoFisher Scientific) in electron counting mode (Astbury Biostructure Laboratories, Leeds, UK). Grids were tilted to 30° to increase the number of side views visualized due to preferential orientation. All spike and Fab complexes were imaged at a nominal magnification of ×75,000 with an object sampling of 1.065 Å/pixel using a 100 μm objective aperture. Supplementary Table 1 contains imaging parameters and Supplementary Fig. 21 shows a representative micrograph from each data collection.

### Image processing

Single-particle image processing was carried out in Relion version 3.0.1 and 3.1.1^48^. Anisotropic image motion was corrected using MOTIONCOR2^49^, and CTFFIND-4.1 was used to estimate the contrast transfer function for each movie^50^. Subsequently, particles were automatically selected using the Laplacian-of-Gaussian picker. Fourier binned (4 × 4) particles were extracted in an 80-pixel box and subjected to a single round of 2D classification, and ‘Junk’ data was subsequently removed from the dataset. For 3D classification of 46C12, 43E6, and 47C9, the UCSF Chimera (version 1.15.0) ‘molmap’ command was used to generate a 50 Å resolution starting model from the OC43 spike model (6OHW)^18^. For 37F1, a starting model was generated ab initio using the particles belonging to the ‘best’ 37F1 2D classes. This was subsequently low pass filtered to 60 Å for 3D classification. For each sample, 100 iterations of a 3D classification were performed with C1 symmetry and particles belonging to the Fab-bound class were re-extracted unbinned in a 320-pixel box. For each data set, iterative rounds of 3D auto-refinement (with C3 symmetry), postprocessing and per-particle CTF refinement were used to account for the stage tilt used during data collection. This was repeated until no further gains in resolution could be achieved. At this point, Bayesian polishing was implemented, and the rounds of refinement were repeated until no further improvement in resolution was observed. For 46C12, 43E6, and 37F1, an additional round of no-alignment 3D classification was performed and particles belonging to the best-resolved class were 3D auto-refined and post-processed. Local resolution estimation, performed in RELION, yielded locally filtered maps. An overview of the data processing pipeline for each sample is shown in Supplementary Figs. 6–8.

### Model building and refinement

The OC43 S trimer model (PDB 6NZK) was used as a starting point for modelling the Fab-bound complexes. The atomic coordinates were fitted into the density maps using the UCSF Chimera (version 1.15.0) “Fit in map” tool^51^. For each of the antibody Fab fragments, a homology model of the variable regions of the heavy chain (HC) and the light chain (LC) were generated using the phyre2 server^52^. These models were then fitted into the corresponding density maps as described for the spike. Iterative rounds of manual fitting in Coot and real space refinement in Phenix (version 1.19.2_4158) were carried out to improve non-ideal rotamers, bond angles, and Ramachandran outliers. To facilitate model building, map sharpening was performed using DeepEMhancer (version 0.13)^53^, as implemented in COSMIC2. The local-resolution filtered maps, generated in Relion, were used for real space refinement, and secondary structure and NCS restraints were imposed throughout. Validation was carried out using Molprobity (version 4.02-528)^54^ (general/protein) and EMringer (version 1.0.0)^55^. Data collection, image processing, and refinement information can be found in Supplementary Table 1 and Supplementary Figs. 6–8. All structure calculations and model building were performed using software compiled by SBGrid.

### Analysis and visualization

PDBePISA (version 1.52) and LigPlot+ 2.2^56^ were used to identify spike residues interacting with the 46C12, 43E6 and 47C9 antibody Fab fragments^57^. The UCSF Chimera (version 1.15.0) “MatchMaker” tool was used to obtain RMSD values, using default settings. Figures were generated using UCSF Chimera (version 1.15.0)^51^ and UCSF ChimeraX (version 1.2.5)^58^.

### Hydrogen deuterium exchange mass spectrometry

For the initial peptide identifications, 3 µl of OC43 S1 (99 pmol) expressed from HEK293 GnTI−/− cells were diluted in 38 µl of 100 mM Tris, 150 mM NaCl, pH 8.0 and mixed with 20 µl quench solution consisting of 6 M Urea and 300 mM TCEP, pH 2.5. Directly after mixing the sample with quench solution, the complete volume of the sample was manually injected into a 50 µl sample loop of nanoACQUITY UPLC System with HDX technology (Waters Corp., USA). The protein was loaded onto the immobilized pepsin column (2.1 × 30 mm, NovaBioAssays, USA) for digestion (20˚C) followed by in-line trapping (0.5 °C) of the formed peptic peptides on the Waters Van Guard^TM^ BEH C18 trap column (300 Å, 1.7 µm, 2.1 × 5 mm) for 3 min at 125 µl/min. Peptic peptides were separated with Waters Acquity UPLC BEH C18 analytical column (1.7 µm, 1.0 × 100 mm) with 15-min gradient from 8 to 95% B, where A is 0.1% formic acid and B is 0.1% formic acid in acetonitrile. Separated peptides were analyzed with XEVO G2 mass spectrometer (Waters Corp., USA) using MSE data acquisition with 6 V fixed collision energy during the low energy scan and 10–35 V ramped collision energy applied during the high energy scan. Cone voltage was set to 40 V. The data was acquired in resolution mode within 50–2000 *m/z* range. The peptide identification measurements were performed in triplicate. Peptides were identified with Waters PLGS 3.0.1 software (digestion was set to non-specific, methionine oxidation, N-glycans, and O-glycans were set as variable modifications) and processed with Waters DynamX 3.0 (minimum intensity was set to 1000, minimum four amino acids were considered as a peptide, peptide was present in two out of three PLGS files). After this initial filtering peptides were visually inspected and selected for spectrum quality, omitting peptides with wrongly assigned charge states and interfering signals. The final OC43 S1 sequence coverage was 76% covered by 127 peptic peptides. These peptides were followed in hydrogen deuterium exchange reactions. In each hydrogen deuterium exchange reaction, 2 µl of OC43 S1 (66 pmol) were incubated either with 1 µl of buffer (100 mM Tris, 150 mM NaCl, pH 8.0) or with 1 µl of Fabs (200 pmol of either 56E10, 45B9 or 65A11 Fabs) for 1 h at room temperature to form S1-Fab complexes. These mixtures were diluted 13-fold into 100 mM Tris, 150 mM NaCl, pD 8.0, 95% D_2_O and quenched with 20 µl of 6 M Urea, 300 mM TCEP, pH 2.5 at 10 s, 1 min, 60 min, and 240 min time points. All measurements were performed in triplicates. The deuterium uptake of the peptides from unbound OC43 was compared to the deuterium uptake of peptides originating from S1-Fab complexes in DynamX 3.0. No back-exchange corrections were conducted due to identical conditions for all performed experiments and deuterium levels were given as a relative deuterium uptake. The statistical analysis of deuterium uptake differences was performed with Deuteros 2.0^59^.

### Maximum likelihood phylogeny

Full-length OC43 S genes deposited until July 2021 were retrieved from GenBank. Multiple sequence alignment was performed using the CLUSTAL W program. The alignment was refined by excluding identical- and near-identical sequences collected in the same year and/or from the same geographic location and by discarding phylogenetic outliers. Rooted phylogenetic trees were constructed using MEGA X software^60^ from a final collection of 124 North American/Eurasion sequences (see Supplementary Table 2) by using the neighbor-joining method with bovine coronavirus S as outgroup. Bootstrapping was performed for 1000 replicates, and evolutionary distances were computed using the Kimura 2-parameter method. Branches were re-ordered from top to bottom based on collection date with the year of isolation indicated by color-coding in a sidebar to visualize S evolution among and within distinct S lineages.

### Reporting summary

Further information on research design is available in the Nature Research Reporting Summary linked to this article.

## Supplementary information

Supplementary information

Reporting Summary

## Data Availability

Data underlying Figs. 1c, 2a, b, 3d, e, 5c, 6b, c, 7c, d, Supplementary Figs 1d-e, 5, 10, 14, 16, 17 and 20 are provided as Source Data files in a publicly accessible repository (https://figshare.com/s/84afe2bace94f17d73ef). PDB files of OC43 spike protein (PDB ID: 6NZK and 6OHW) were downloaded from NCBI database (https://www.ncbi.nlm.nih.gov/). Spike protein sequences used in this study were downloaded from NCBI database (https://www.ncbi.nlm.nih.gov/) (See Supplementary Table 2 for the accession numbers). Sequences of the monoclonal antibodies characterized here are available from GenBank under the following accession numbers: OK245435, OK245436, OK245437, OK245438, OK245439, OK245440, OK245441, OK245442, OK245443, OK245444, OK245445, OK245446, OK245447, OK245448, OK245449, OK245450, OK245451 and OK245452. The accession numbers for the cryo-EM structures of HCoV-OC43 S with 46C12/43E6/47C9 Fab reported in this paper is PDB ID 7PNM, 7PNQ and 7PO5 respectively. Source data are provided with this paper.
