# Supplementary material for: Antigenic structure of the human coronavirus OC43 spike reveals exposed and occluded neutralizing epitopes
Source: Nat Commun. 2022 May 25;13:2921. doi: 10.1038/s41467-022-30658-0 (PMC9132891; doi:10.1038/s41467-022-30658-0)
Supplement: Supplementary file 1 — Supplementary information [file 41467_2022_30658_MOESM1_ESM.pdf]

Supplementary Information

Antigenic structure of the Human Coronavirus OC43 Spike reveals Exposed and Occluded Neutralizing Epitopes

Chunyan Wang, Emma L. Hesketh, Tatiana Shamorkina, Wentao Li, Peter J. Franken, Dubravka Drabek, Rien van Haperen, Sarah Townend, Frank J.M. van Kuppeveld, Frank Grosveld, Neil A. Ranson, Joost Snijder, Raoul J. de Groot, Daniel L. Hurdiss and Berend-Jan Bosch

Supplementary Table 1 Cryo-EM data collection, refinement and validation statistics.

	OC43-S + 46C12 (EMDB-13549) (PDB 7PNM)	OC43-S + 43E6 (EMDB-13550) (PDB 7PNQ)	OC43-S + 47C9 (EMDB-13563) (PDB 7PO5)	OC43-S + 37F1 (EMDB-13564)
**Data collection and processing**				
Magnification	75,000x	75,000x	75,000x	75,000x
Voltage (kV)	300	300	300	300
Electron exposure (e–/Å^2)	52	52	52	52
Defocus range (μm)	0.8-2.6	0.8-2.6	0.8-2.6	0.8-2.6
Pixel size (Å)	1.065	1.065	1.065	1.065
Symmetry imposed	C3	C3	C3	C3
Initial particle images (no.)	482,414	349,851	144,035	635,273
Final particle images (no.)	76,638	53,157	45,798	23,452
Map resolution (Å)	3.7	3.7	3.9	4.4
FSC threshold	0.143	0.143	0.143	0.143
Map resolution range (Å)	3.5-9.5	3.5-10.1	3.7-7.6	3.9-14.5
**Refinement**				
Initial model used (PDB code)	6NZK	6NZK	6NZK	-
Model resolution (Å)	3.9	3.7	4	-
FSC threshold	0.5	0.5	0.5	-
Map sharpening *B* factor (Å^2)	-139	-150	-180	-
Model composition				
Non-hydrogen atoms	33,540	32,925	32,934	-
Protein residues	4,224	4,218	4,218	-
Ligands	48	15	15	-
B factors (Å^2)				
Protein	68	75.68	36.21	-
Ligand (Glycans)	89.05	98.88	70.37	-
R.m.s. deviations				
Bond lengths (Å)	0.003	0.003	0.003	-
Bond angles (°)	0.741	0.650	0.655	-
Validation				
MolProbity score	1.5	1.26	1.34	-
Clashscore	4.49	2.92	3.57	-
Poor rotamers (%)	0.14	0.03	0.03	-
Ramachandran plot				
Favored (%)	96	96.93	96.43	-
Allowed (%)	4	3	3.57	-
Disallowed (%)	0	0.07	0	-

Supplementary Table 2 OC43 spike gene sequences used to calculate the phylogenetic tree shown in Fig. 7a.

Genbank ID:	spike ID:	strain:	Country of isolation:	Year of isolation:
MZ450972.1	QXL74886.1	OC43/Seattle/USA/20959/2021	USA	2021
MT118683.1	QQY99297.1	2019_0522	USA	2019
MN306053.1	QEG03794.1	HCoV_OC43/Seattle/USA/SC9430/2018	USA	2018
MT118692.1	QQY99373.1	2019_1540	USA	2019
MT118673.1	QQY99219.1	2018_9142	USA	2018
MW532119.1	QRK03922.1	OC43/China/10/2017	China	2017
OK245434*	OK245433	NL/2019	Netherlands	2019
MW532118.1	QRK03912.1	OC43/China/09/2017	China	2017
MT118667.1	QQY99171.1	2017_1822	USA	2017
MG197723.1	AXX83381.1	HZ-459	China	2016
KY967358.1	ARK08651.1	HCoV_OC43/Seattle/USA/SC2770/2015	USA	2015
KU745535.1	ANZ78836.1	14007/2014	China	2014
KF923904.1	AIV41885.1	12694/2012	China	2012
KX538964.1	AQN78656.1	MY-U002/12	Malaysia	2012
MG197710.1	AXX83303.1	BJ-124	China	2015
KU745545.1	ANZ78846.1	4452A/2015	China	2015
KU745548.1	ANZ78849.1	SZ14014d3/2014	China	2014
LC315647.1	BBA20976.1	Tokyo/SGH-61/2014	Japan	2014
MF374983.2	AVR40342.1	HCoV-OC43/USA/TCNP_0070/2016	USA	2016
KX538966.1	AQN78672.1	MY-U236/12	Malaysia	2012
KY684759.1	ARA15421.1	HCoV_OC43/Seattle/USA/SC2269/2016	USA	2016
KY967356.1	ARK08635.1	HCoV_OC43/Seattle/USA/SC2924/2015	USA	2015
KU745539.1	ANZ78840.1	4086A/2014	China	2014
KX538978.1	AQN78768.1	MY-U1758/13	Malaysia	2013
KF923902.1	AIV41873.1	12689/2012	China	2012
KF572831.1	AIL49511.1	12689_12	China	2012
KF963242.1	AIX10761.1	HCoV-OC43/FRA_EPI/Caen/2011/13	France	2011
KF572829.1	AIL49509.1	11930_11	China	2011
KF963244.1	AIX10763.1	HCoV-OC43/FRA_EPI/Caen/2013/15	France	2013
KF963243.1	AIX10762.1	HCoV-OC43/FRA_EPI/Caen/2012/14	France	2012
KX538974.1	AQN78736.1	MY-U945/12	Malaysia	2012
KF923903.1	AIV41879.1	12691/201	China	2012
KX538975.1	AQN78744.1	MY-U1024/12	Malaysia	2012
KX344031.1	AOL02453.1	LRTI_238	Mexico	2011
KF963240.1	AIX10759.1	HCoV-OC43/FRA_EPI/Caen/2009/11	France	2009
KF572840.1	AIL49520.1	3098A_12	China	2012
KJ958219.1	AIX09807.1	LY342	China	2011
AIL49495.1	AIL49518.1	2134A_10	China	2010
KF572821.1	AIL49501.1	10108_10	China	2010
KF572867.1	AIL49547.1	8375_09	China	2009
KF923893.1	AIV41819.1	2151A/2010	China	2010
KF572872.1	AIL49552.1	978A_08	China	2008
KF572815.1	AIL49495.1	1489A_09	China	2009
KY674920.1	ARB07433.1	N09-595B	China	2016
KF572868.1	AIL49548.1	892A_08	China	2008
KF923916.1	AIV41957.1	5519/2007	China	2007
KF572862.1	AIL49542.1	5625_07	China	2007
KF572820.1	AIL49500.1	079A_07	China	2007
MN488635.1	QHB49099.1	4310800120_seq2	Netherlands	2008
KF572843.1	AIL49523.1	4954_07	China	2007
KF923908.1	AIV41909.1	5414/2007	China	2007
KF572854.1	AIL49534.1	5484_07	China	2007
KF572857.1	AIL49537.1	5517_07	China	2007
KF572818.1	AIL49498.1	3647_06	China	2006
KF923905.1	AIV41891.1	229/2005	China	2005
JN129834.1	AEN19358.1	HK04-01	Hong Kong	2004
MN488634.1	QHB49098.1	4310600253_seq2	Netherlands	2006
JN129835.1	AEN19366.1	HK04-02	Hong Kong	2004
MN488633.1	QHB49097.1	4310600157_seq2	Netherlands	2006
KF963235.1	AIX10754.1	HCoV-OC43/FRA_EPI/Caen/2004/06	France	2004
AY903455.1*	AAX84792.1	34364 Belgium 2004	Belgium	2004
KU745542.1	ANZ78843.1	4446A/2015	China	2015
KF923895.1	AIV41831.1	10285/2010	China	2010
KF963231.1	AIX10750.1	HCoV-OC43/FRA_EPI/Caen/2001/02	France	2001
KF530068.1	AGT51491.1	OC43/human/USA/007-11/2000	USA	2000
KF530081.1	AGT51620.1	OC43/human/USA/991-5/1999	USA	1999
KF530070.1	AGT51511.1	OC43/human/USA/991-19/1999	USA	1999
KF530069.1	AGT51501.1	OC43/human/USA/982-4/1998	USA	1998
KF530099.1	AGT51800.1	OC43/human/USA/971-5/1997	USA	1997
KF530063.1	AGT51441.1	OC43/human/USA/9612-48/1996	USA	1996
KF530098.1	AGT51790.1	OC43/human/USA/965-6/1996	USA	1996
KF530084.1	AGT51650.1	OC43/human/USA/951-18/1995	USA	1995
KF530071.1	AGT51521.1	OC43/human/USA/925-1/1992	USA	1992
KF530076.1	AGT51570.1	OC43/human/USA/911-11/1991	USA	1991
KF530088.1*	AGT51690.1	OC43/human/USA/901-54/1990	USA	1990
KF530079.1	AGT51600.1	OC43/human/USA/913-29/1991	USA	1991
OK245433*	OK245433	NL/2004	Netherlands	2004
MN310476.1	QEG03803.1	HCoV_OC43/Seattle/USA/SC9428/2018	USA	2018
MN306043.1	QEG03776.1	HCoV_OC43/Seattle/USA/SC0841/2019	USA	2019
MW532108.1	QRK03812.1	OC43/China/07/201	China	2017
MT501650.1	QLC35792.1	cyb-BetaCoV/2019	China	2019
KU745540.1	ANZ78841.1	4400A/2015	China	2015
MW532109.1	QRK03822.1	OC43/China/12/2018	China	2018
MG197719.1	AXX83357.1	YC-67	China	2015
KU745541.1	ANZ78842.1	4436A/2015	China	2015
KF572813.1	AIL49493.1	3184A_12	China	2012
MG197709.1	AXX83297.1	BJ-112	China	2015
MT118671.1	QQY99202.1	2018_8596	USA	2018
KY967360.1	ARK08670.1	HCoV_OC43/Seattle/USA/SC2476/2015	USA	2015
MF314143.1*	AVQ05264.1	HCoV-OC43/USA/ACRI_0052/2016	USA	2016
KU131570.1	AMK59677.1	HCoV-OC43/UK/London/2011	UK	2011
LC315649.1	BBA20982.1	Tokyo/SGH-65/2016	Japan	2016
KF963241.1	AIX10760.1	HCoV-OC43/FRA_EPI/Caen/2010/12	France	2010
KF572812.1	AIL49492.1	3074A_12	China	2012
KF572811.1	AIL49491.1	2941A_11	China	2011
KF572804.1	AIL49484.1	1783A_10	China	2010
KF572814.1	AIL49494.1	3194A_12	China	2012
KF963238.1	AIX10757.1	HCoV-OC43/FRA_EPI/Caen/2007/09	France	2007
KF963239.1	AIX10758.1	HCoV-OC43/FRA_EPI/Caen/2008/10	France	2008
KF572805.1	AIL49485.1	1908A_10	China	2010
KF963237.1	AIX10756.1	HCoV-OC43/FRA_EPI/Caen/2006/08	France	2006
AY903454.1	AAX84791.1	89996 Belgium 2003	Belgium	2003
AY903459.1	AAX85669.1	87309 Belgium 2003	Belgium	2003
KF963234.1	AIX10753.1	HCoV-OC43/FRA_EPI/Caen/2003/05	France	2003
AY903457.1	AAX84794.1	37767 Belgium 2003	Belgium	2003
AY903456.1	AAX84793.1	84020 Belgium 2003	Belgium	2003
Z21849	CAA79896.1	HCV-OC43	?	?
AY391777.1*	AAR01015.1	ATCC VR-759	USA	1967
KF963236.1	AIX10755.1	HCoV-OC43/FRA_EPI/Caen/2005/07	France	2005
KF963233.1	AIX10752.1	HCoV-OC43/FRA_EPI/Caen/2002/04	France	2002
KF963230.1	AIX10749.1	HCoV-OC43/FRA_EPI/Caen/2001/01	France	2001
KF530092.1	AGT51730.1	OC43/human/USA/008-5/2000	USA	2000
KF530072.1	AGT51531.1	OC43/human/USA/9712-13/1997	USA	1997
KF530080.1	AGT51610.1	OC43/human/USA/9712-31/1997	USA	1997
KF530078.1	AGT51590.1	OC43/human/USA/9612-29/1996	USA	1996
KF530062.1	AGT51431.1	OC43/human/USA/952-23/1995	USA	1995
KF530074.1	AGT51551.1	OC43/human/USA/9212-33/1992	USA	1992
KF530065.1*	AGT51461.1	OC43/human/USA/901-41/1990	USA	1990
KF530073.1	AGT51541.1	OC43/human/USA/8912-37/1989	USA	1989
KF530085.1	AGT51660.1	OC43/human/USA/871-25/1987	USA	1987
KF530077.1	AGT51580.1	OC43/human/USA/873-16/1987	USA	1987
KF530060.1	AGT51412.1	OC43/human/USA/851-15/1985	USA	1985
KF530087.1	AGT51680.1	OC43/human/USA/873-6/1987	USA	1987
KF530093.1	AGT51740.1	OC43/human/USA/832-27/1983	USA	1983

* GenBank ID order corresponds to the positions of S types in the phylogenetic tree from top to bottom (Fig. 7a). S types used to assess antibody binding breadth (Fig. 7c) are highlighted in red.

Supplementary Fig. 1

a

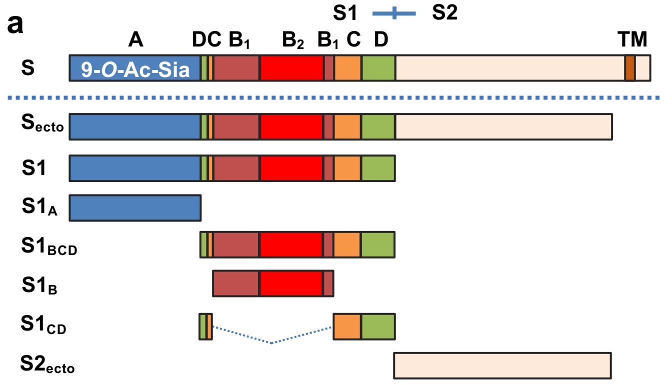

b

ELISA-reactivity hybridoma supernatants (5 fold diluted) to OC43 spike proteins (OD_{450} nm)

No.	Hybridoma	Secto	S1	S1A	S1BCD	S1B	S1CD	S2ecto
1	54B5	2.038	3.066	3.631	0.061	0.053	0.067	0.048
2	60E5	2.213	3.36	3.775	0.048	0.042	0.053	0.043
3	43H8	1.901	2.577	3.169	0.387	0.177	0.062	0.041
4	46C12	3.533	3.869	4.021	0.08	0.053	0.079	0.051
5	26E12	3.073	3.736	3.824	0.046	0.051	0.054	0.039
6	27G3	3.401	3.803	3.832	0.048	0.051	0.051	0.04
7	41F12	3.556	3.955	3.977	0.066	0.061	0.093	0.084
8	47H1	3.348	3.871	3.915	0.092	0.075	0.069	0.043
9	62A12	3.692	3.989	4.086	0.115	0.081	0.055	0.05
10	66B1	2.383	2.82	3.085	0.133	0.09	0.084	0.063
11	46G6	3.993	4.128	0.083	3.854	0.094	0.156	0.062
12	43B8	3.555	3.986	0.06	3.865	0.047	0.059	0.038
13	44H8	3.591	4.082	0.074	3.908	0.067	0.056	0.045
14	44B8	1.631	2.055	0.056	2.506	0.043	0.082	0.044
15	45D10	3.095	3.399	0.08	3.438	0.072	0.071	0.047
16	45B9	2.801	3.478	0.073	3.508	2.46	0.086	0.046
17	37F1	2.531	3.412	0.073	3.241	1.995	0.069	0.113
18	38E12	3.347	3.934	0.069	3.907	3.584	0.07	0.077
19	42A7	3.635	4.202	0.074	4.058	2.052	0.065	0.041
20	43E6	3.996	4.23	0.065	4.093	3.976	0.061	0.041
21	23C11	4.111	4.249	0.068	3.953	4.027	0.064	0.041
22	24A2	4.251	4.338	0.06	3.82	4.242	0.061	0.044
23	24E1	3.742	4.107	0.082	4.014	4.233	0.084	0.046
24	25D4	2.887	3.391	0.062	3.125	2.742	0.049	0.048
25	21E7	3.782	4.097	0.075	3.886	1.235	0.05	0.04
26	23C6	3.904	4.178	0.085	4.147	4.014	0.082	0.041
27	40D11	3.247	3.633	0.07	3.427	3.034	0.053	0.05
28	44H2	0.882	1.135	0.066	0.959	0.447	0.048	0.045
29	44G2	3.971	4.231	0.066	4.064	1.403	0.057	0.039
30	47C9	3.926	4.17	0.072	3.964	3.877	0.047	0.044
31	47C10	0.854	1.094	0.104	0.911	0.454	0.053	0.045
32	38C5	3.089	4.164	0.081	4.091	4.11	0.06	0.047
33	56E10	3.984	4.13	0.095	3.838	3.109	0.057	0.044
34	65A11	3.773	4.045	0.075	3.864	3.727	0.062	0.05
35	42D10	3.67	4.076	0.099	3.833	3.616	0.046	0.048
36	36E2	4.211	4.243	0.08	4.083	4.173	0.054	0.055
37	49C6	3.889	4.211	0.707	3.76	3.889	0.068	0.05
38	38F1	2.93	3.629	0.096	3.347	2.165	0.059	0.043
39	1B2	3.566	4.042	0.116	3.768	3.21	0.053	0.042
40	35E2	1.076	1.511	0.099	1.299	0.786	0.064	0.05
41	62C3	2.418	2.828	0.094	2.52	1.823	0.078	0.048
42	37E6	3.73	3.984	0.079	3.767	1.226	0.06	0.045
43	63A5	3.701	4.071	0.066	3.935	2.444	0.055	0.046
44	23G5	3.607	3.871	0.066	3.798	3.277	0.057	0.052
45	74F12	3.818	3.994	0.067	3.812	3.497	0.058	0.058
46	41B5	1.36	3.375	0.063	3.571	4.018	0.06	0.066
47	35E12	3.441	3.81	0.063	3.483	0.059	2.485	0.091
48	39H4	2.823	3.152	0.062	3.07	0.062	0.623	0.096
49	49A11	3.528	3.684	0.059	3.34	0.033	2.56	0.042
50	50A6	3.955	4.079	0.061	3.646	0.044	3.502	0.041
51	49H11	4.015	4.103	0.07	3.72	0.074	3.646	0.042
52	34G2	3.809	4.025	0.065	3.731	0.072	2.193	0.043
53	41A1	2.81	3.03	0.067	2.785	0.055	1.628	0.041
54	41H10	3.423	3.609	0.117	3.478	0.04	2.521	0.04
55	49E11	3.998	4.131	0.09	3.858	0.08	3.594	0.057
56	49B4	3.934	4.093	0.059	3.82	0.058	3.212	0.041
57	44C11	3.95	4.112	0.077	3.842	0.071	3.473	0.052
58	50C12	2.159	2.45	0.121	2.523	0.062	1.243	0.044
59	44G3	3.484	3.663	0.073	3.488	0.064	3.571	0.047
60	48E10	2.307	2.789	0.053	2.756	0.081	1.884	0.079
61	56G3	3.979	4.069	0.085	3.871	0.039	3.686	0.043
62	5A11	2.409	0.081	0.099	0.069	0.067	0.088	0.526
63	8C9	0.453	0.052	0.073	0.048	0.09	0.052	0.191
64	12B1	2.758	0.074	0.092	0.063	0.088	0.078	2.04
65	12E3	0.578	0.063	0.074	0.062	0.075	0.073	0.175
66	13D10	0.446	0.079	0.09	0.079	0.116	0.095	0.245
67	14B10	0.524	0.081	0.096	0.073	0.076	0.086	0.195
68	22C5	1.854	0.077	0.083	0.049	0.093	0.07	1.11
69	31H1	2.348	0.065	0.076	0.057	0.05	0.065	1.378
70	32F2	1.336	0.117	0.131	0.109	0.071	0.123	1.24
71	5G9	0.779	0.153	0.178	0.146	0.174	0.204	0.437
72	43F11	2.825	0.085	0.145	0.072	0.046	0.088	2.68
73	47C4	2.241	0.176	0.108	0.14	0.074	0.065	1.635
74	34H2	1.945	0.101	0.079	0.114	0.097	0.074	1.147
75	5H1	0.947	0.085	0.091	0.073	0.067	0.094	0.597

c

OC43 spike domain specificity	# hyb. sups.
S1A	10
S1B	31
S1CD	15
S1BCD, but not S1B or S1CD	5
S2ecto	14
Total	75

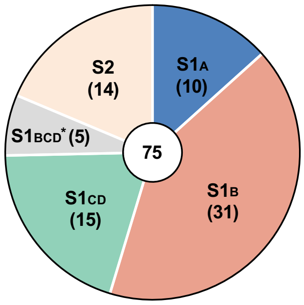

d

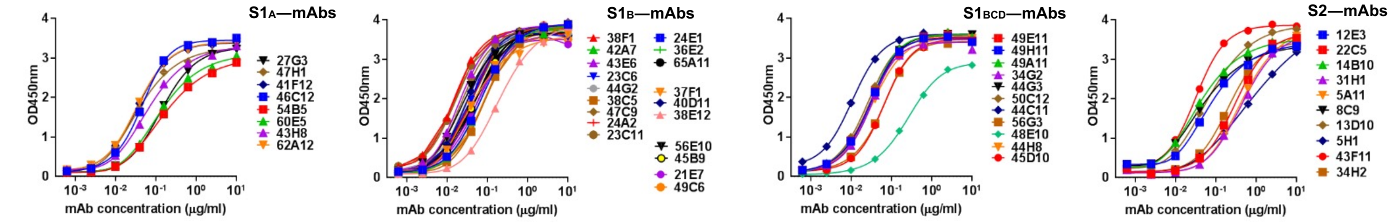

e

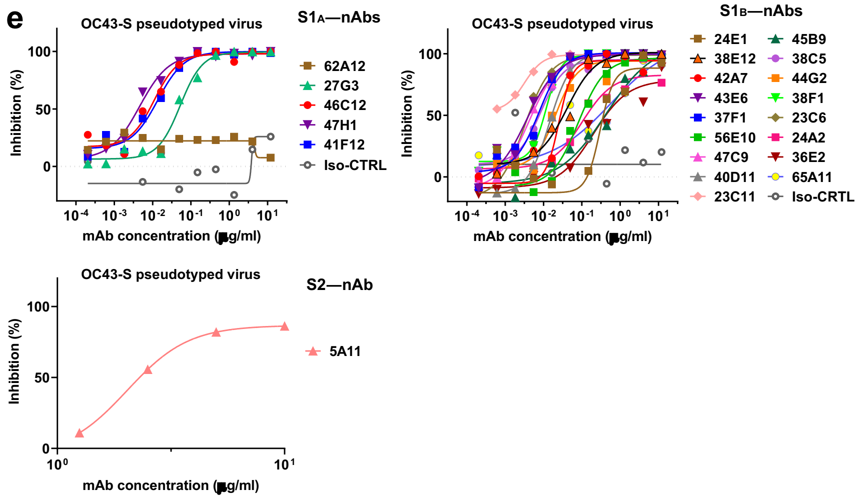

f

mAb 2 \ mAb 1		38F1	24E1	40D11	56E10	41F12
Anti-S1B	38F1	0				
	24E1	0.55	0			
	38E12	0.65	0.65	0		
	42A7	0				
	43E6	0				
	37F1	0.86	0.8	0		
	56E10	0.4	0.45	0.3	0	
	23C6	0				
	44G2	0				
	38C5	0				
	47C9	0				
	40D11	0.8	0.8	0		
	45B9	0.6	0.4	0.3	0	
	24A2	0				
	36E2	0.5	0			
	23C11	0.05				
	65A11		0			
Anti-S1A	41F12					0
	27G3					0
	47H1					0
	46C12					0

Supplementary Fig. 1 Characterization of OC43 S-reactive H2L2 antibodies. a, Upper part: Overall topology of OC43 spike (S) protein monomer. The S subunits (S1 and S2), S1 domains (A through D), transmembrane domain (TM) and 9-*O*-Ac-Sia receptor binding function are indicated. Lower part: recombinant soluble OC43 S antigens used for epitope mapping, including S ectodomain (S_{ecto}), S1 and S1 variants ($S1_A$, $S1_{BCD}$, $S1_B$ and $S1_{CD}$) and the ectodomain of S2 subunit ($S2_{ecto}$). S_{ecto} contains a mutation in the furin cleavage site at the S1/S2 junction, S_{ecto} and $S2_{ecto}$ were C-terminally fused to a GCN4 trimerization motif and a Strep-tag (ST) for purification whereas all S1 domains were C-terminally fused to a human IgG1-Fc purification tag (not shown in figure). **b,** Heatmap showing the ELISA-reactivity of antibodies in H2L2 hybridoma supernatants to different types of OC43 S proteins, including OC43 S_{ecto}, S1, $S1_A$, $S1_{BCD}$, $S1_B$, $S1_{CD}$ and $S2_{ecto}$. The ELISA OD_{450nm} values of hybridoma supernatants (1:5 dilution) for the different antigens are shown and signal intensities are visualized range from red to green indicating high to low OD values, respectively. Antibodies of hybridomas that were successfully subcloned are colored in red. **c,** Pie chart viewing the number of in total 75 OC43 S-specific H2L2 hybridoma supernatants that bind to the indicated OC43 S domains as defined under **b**. $S1_{BCD}^{*}$: mAbs that are reactive to $S1_{BCD}$ but not to the $S1_B$ or $S1_{CD}$. **d,** ELISA binding curves of mAbs to S_{ecto} of OC43. Data from a representative experiment (out of two) are shown. **e,** Neutralizing activity of 22 neutralizing H2L2 mAbs against OC43 S pseudotyped VSV. Symbols represent means of triplicates. **f,** Epitope grouping of OC43 S mAbs using biolayer interferometry. Sensor captured OC43 S_{ecto} was saturated in binding with a given anti-OC43 S mAb1 and then exposed to binding by mAb2. Additional binding of the second antibody indicates the presence of an unoccupied epitope, whereas lack of binding indicates epitope blocking by the first antibody. Binding signal (nm) of mAb2 is indicated in the box.

Supplementary Fig. 2

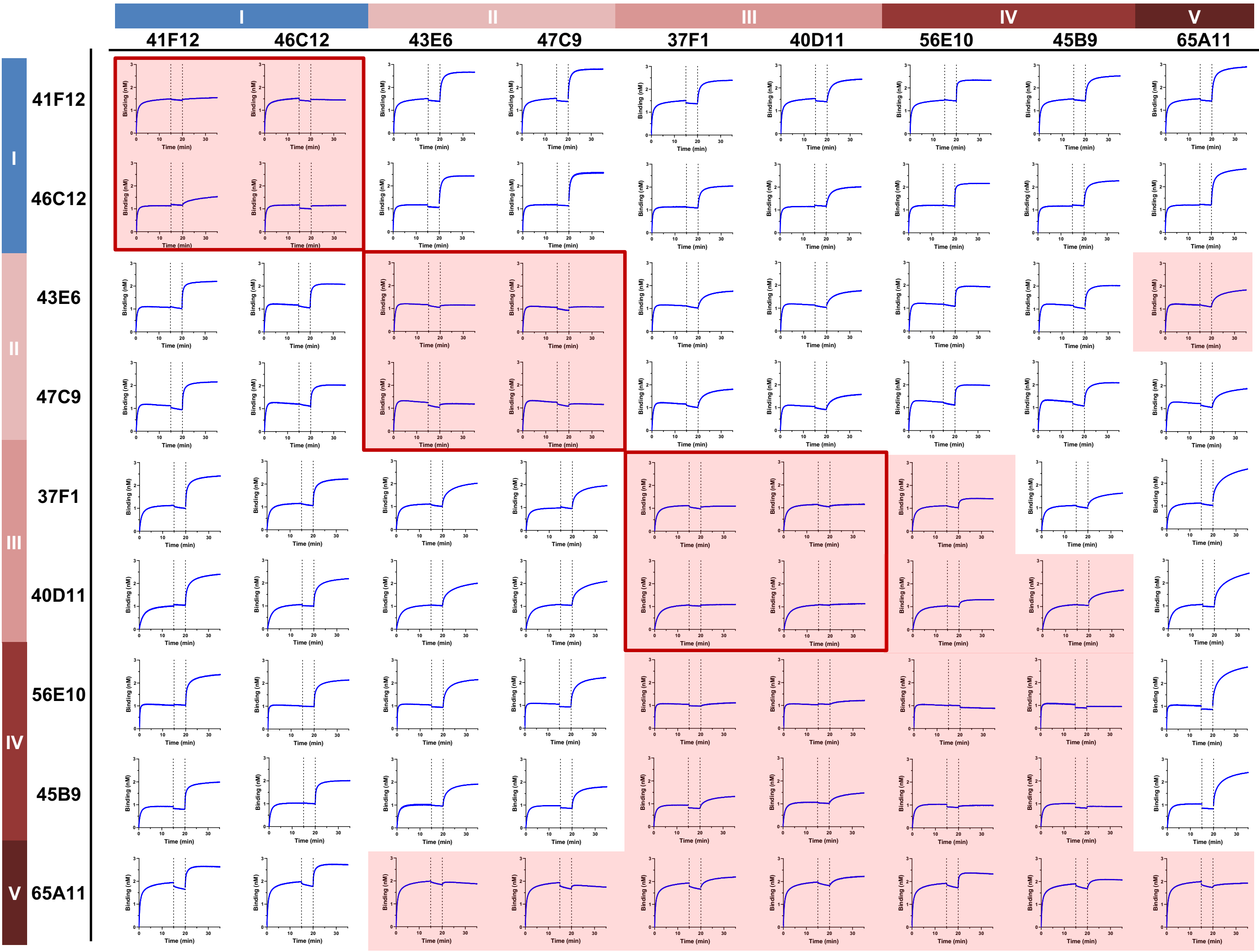

Supplementary Fig. 2 Antibody binding competition analysed by biolayer interferometry. Immobilized OC43 S1 antigen was saturated in binding with a given anti-OC43 S mAb (mAb 1) and then exposed to binding by a second mAb (mAb 2). Additional binding of the second antibody indicates the presence of an unoccupied epitope, whereas lack of binding indicates epitope blocking by mAb1. As a control, the first mAb was also included in the second step to check for self-competition. Colored boxes indicate competition, three mutually exclusive antibody groups (red rectangles, group I-III) were defined as well as two groups (IV and V) with a more miscellaneous binding competition profile. Numbers of antibody groups are indicated on the top and left. A representative experiment (out of two) is shown.

Supplementary Fig. 3

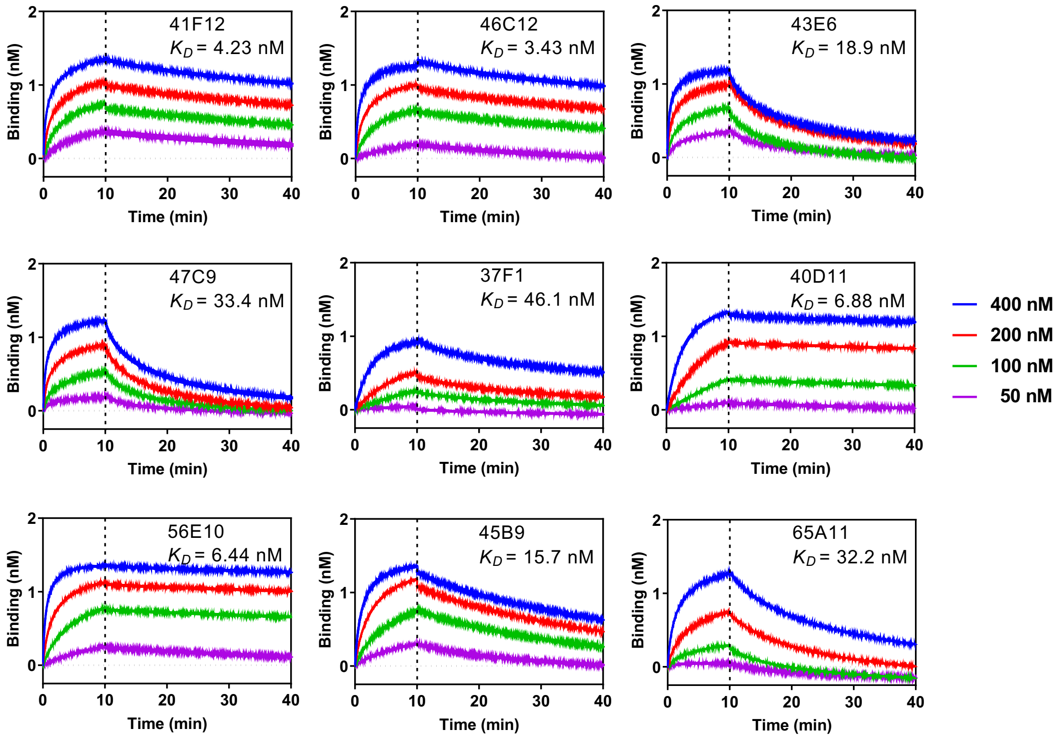

Supplementary Fig. 3 Antibody binding kinetics to OC43 S1. Binding kinetics of S1-directed human mAbs to recombinant OC43 S1 were measured using biolayer interferometry at 25ºC. The binding assay was performed by loading each mAb at optimal concentration (44 nM) to an anti-human Fc biosensor for 10 min. Antigen association step was performed by incubating the sensor with a range of concentrations of monomeric S1 (400-200-100-50 nM) for 10 min, followed by a dissociation step in PBS for 60 min. The kinetics constants were calculated using 1:1 Langmuir binding model on Fortebio Data Analysis 7.0 software. A representative experiment (out of two) is shown.

Supplementary Fig. 4

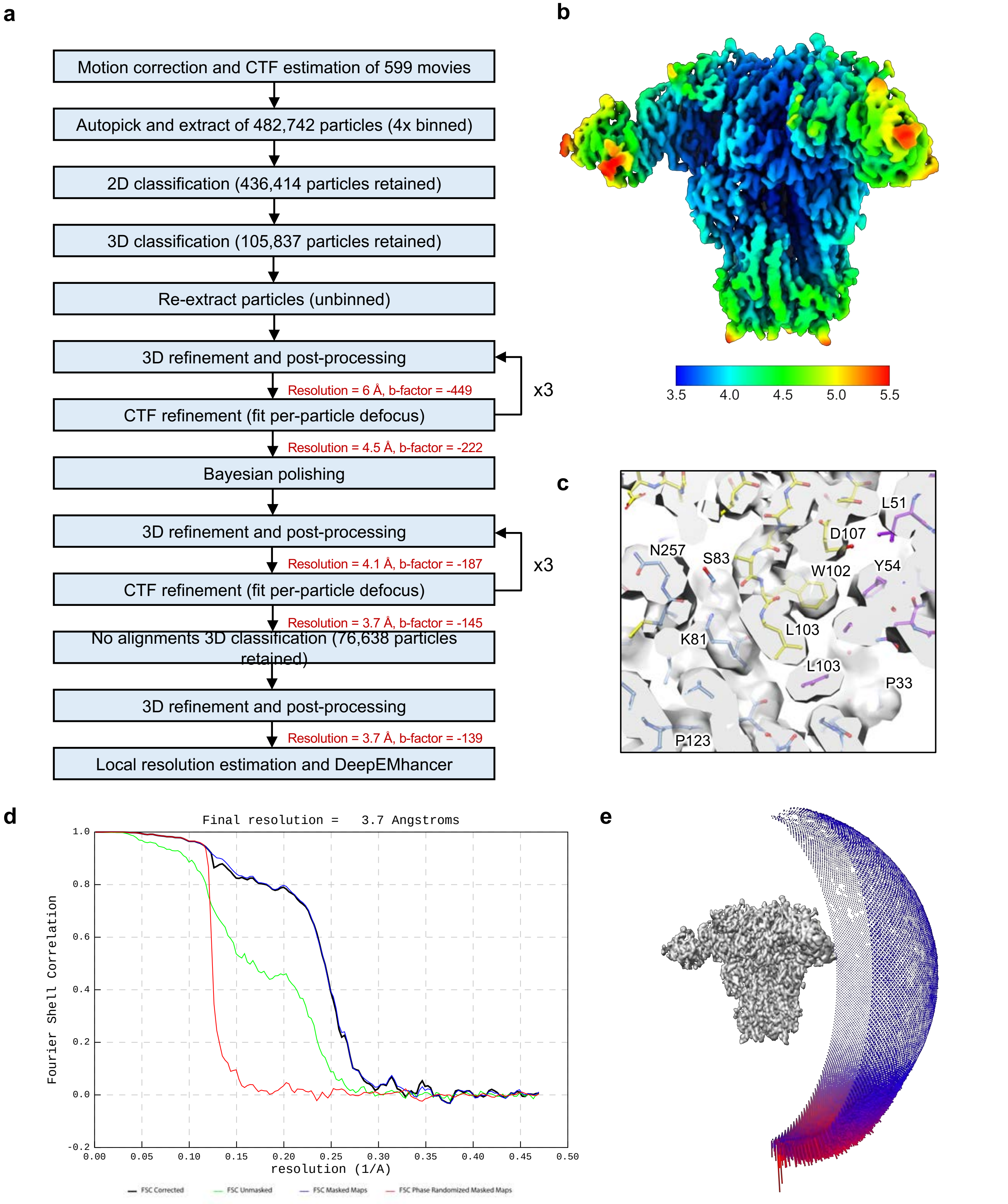

Supplementary Fig. 4 Cryo-EM data processing of OC43-S bound to 46C12. a, Single-particle cryo-EM image processing workflow for the OC43 spike:46C12 data set. **b,** EM density map for the C3 refined complex, colored according to local resolution which was calculated in Relion3.1.1. **c,** EM density for the 46C12 epitope/paratope region. The OC43-S protomer is colored blue and the heavy- and light-chain variable domains are colored yellow and purple, respectively. **d,** Gold-standard Fourier shell correlation (FSC) curve generated from the independent half maps contributing to the 3.7 Å resolution density map. **e,** Angular distribution plot of the final C3 refined OC43 spike:46C12 EM density map.

Supplementary Fig. 5

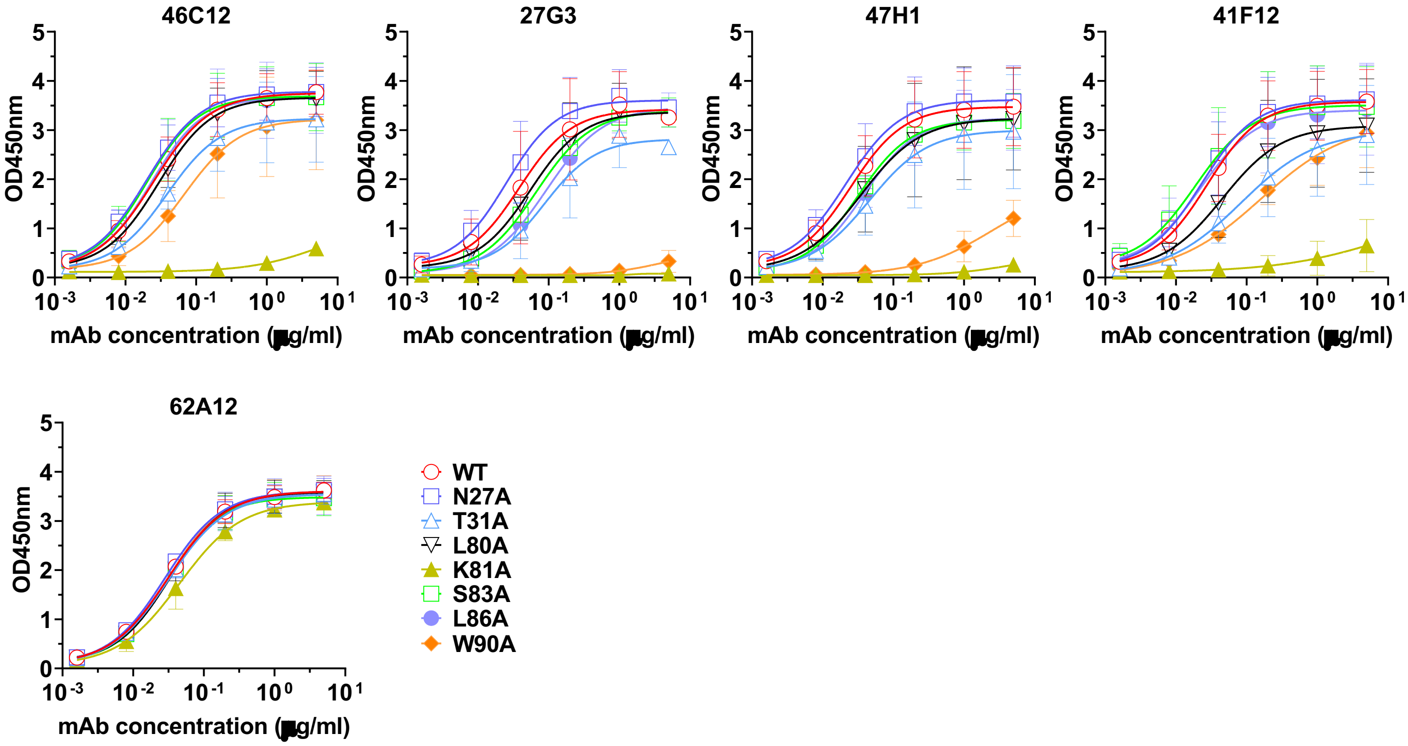

Supplementary Fig. 5 Neutralizing $S1_A$ binding antibodies target the sialoglycan-receptor binding site. Binding of $S1_A$-reactive mAbs to OC43 $S1_A$ with single alanine substitution of 9-*O*-Sia-interacting residues was tested by ELISA. The average ± SD of two biological replicates with four technical replicates is shown.

Supplementary Fig. 6

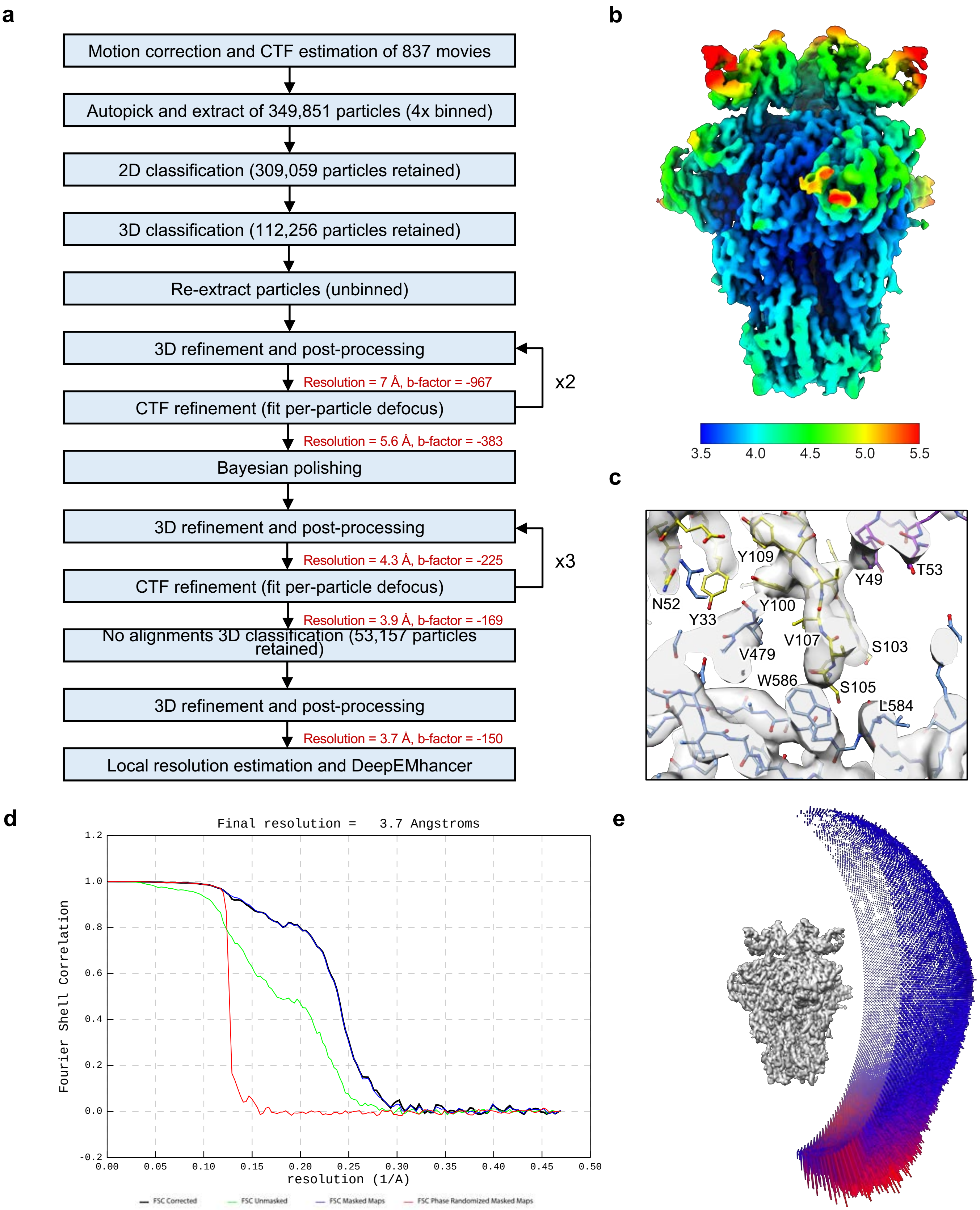

Supplementary Fig. 6 Cryo-EM data processing of OC43-S bound to 43E6. a, Single-particle cryo-EM image processing workflow for the OC43 spike:43E6 data set. **b,** EM density map for the C3 refined complex, colored according to local resolution which was calculated in Relion3.1.1. **c,** EM density for the 43E6 epitope/paratope region. The OC43-S protomer is colored blue and the heavy- and light-chain variable domains are colored yellow and purple, respectively. **d,** Gold-standard Fourier shell correlation (FSC) curve generated from the independent half maps contributing to the 3.7 Å resolution density map. **e,** Angular distribution plot of the final C3 refined OC43 spike:43E6 EM density map.

Supplementary Fig. 7

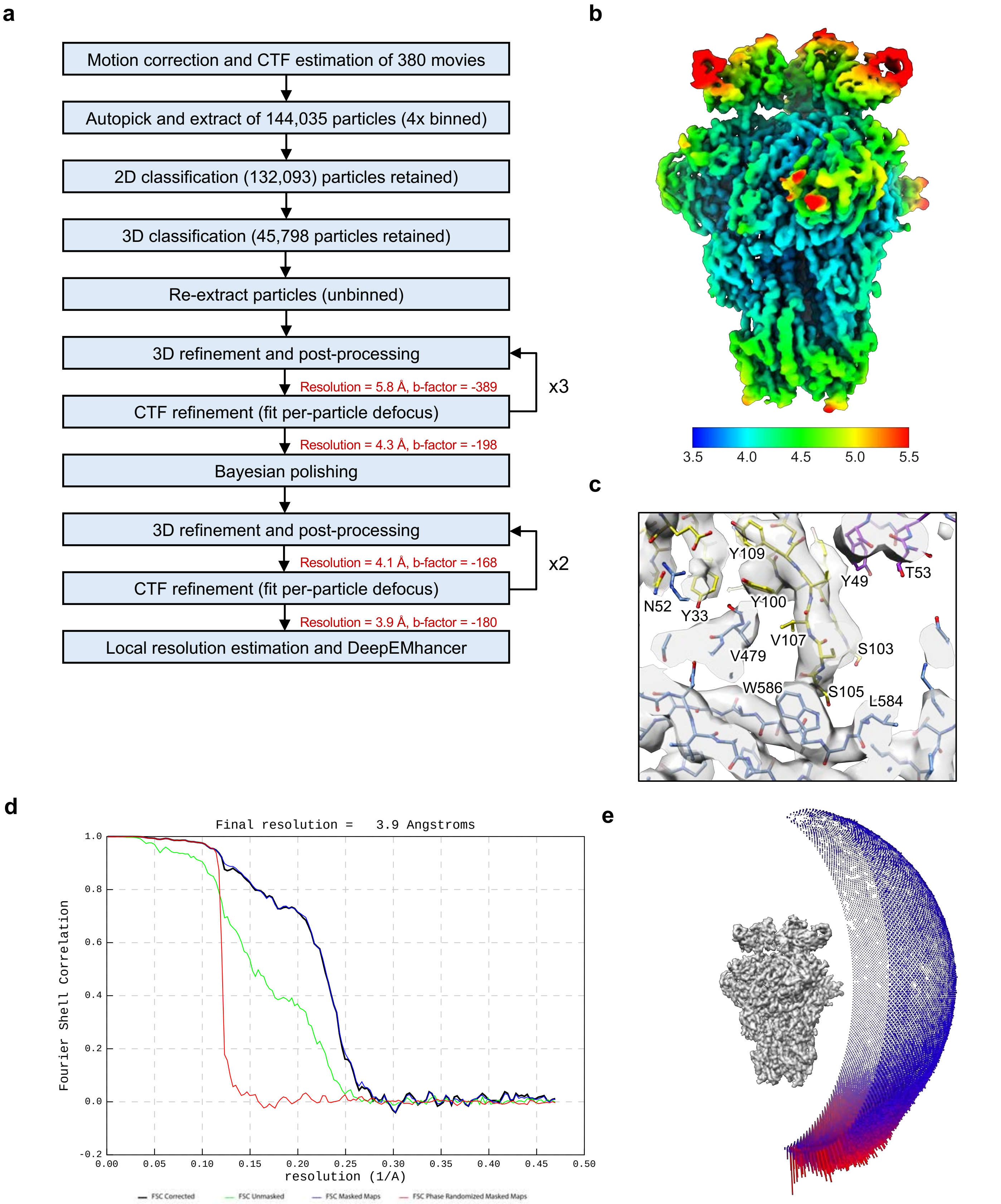

Supplementary Fig. 7 Cryo-EM data processing of OC43-S bound to 47C9. a, Single-particle cryo-EM image processing workflow for the OC43 spike:47C9 data set. **b,** EM density map for the C3 refined complex, colored according to local resolution which was calculated in Relion3.1.1. **c,** EM density for the 47C9 epitope/paratope region. The OC43-S protomer is colored blue and the heavy- and light-chain variable domains are colored yellow and purple, respectively. **d,** Gold-standard Fourier shell correlation (FSC) curve generated from the independent half maps contributing to the 3.9 Å resolution density map. **e,** Angular distribution plot of the final C3 refined OC43 spike:47C9 EM density map.

Supplementary Fig. 8

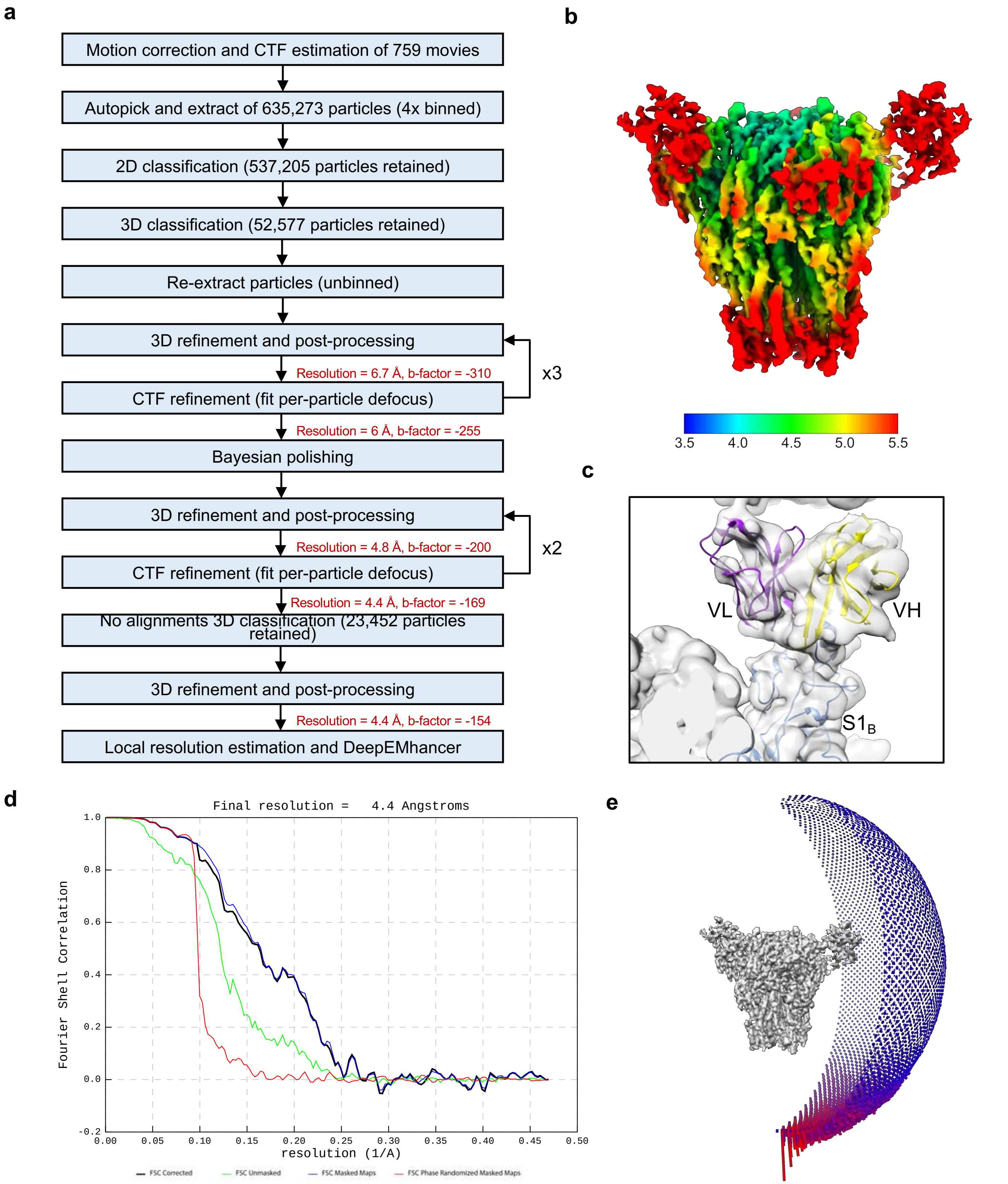

Supplementary Fig. 8 Cryo-EM data processing of OC43-S bound to 37F1. a, Single-particle cryo-EM image processing workflow for the OC43 spike:37F1 data set. **b,** EM density map for the C3 refined complex, colored according to local resolution which was calculated in Relion3.1.1. **c,** EM density for the 37F1 epitope/paratope region. The OC43-S protomer is colored blue and the heavy- and light-chain variable domains are colored yellow and purple, respectively. **d,** Gold-standard Fourier shell correlation (FSC) curve generated from the independent half maps contributing to the 4.4 Å resolution density map. **e,** Angular distribution plot of the final C3 refined OC43 spike:37F1 EM density map.

Supplementary Fig. 9

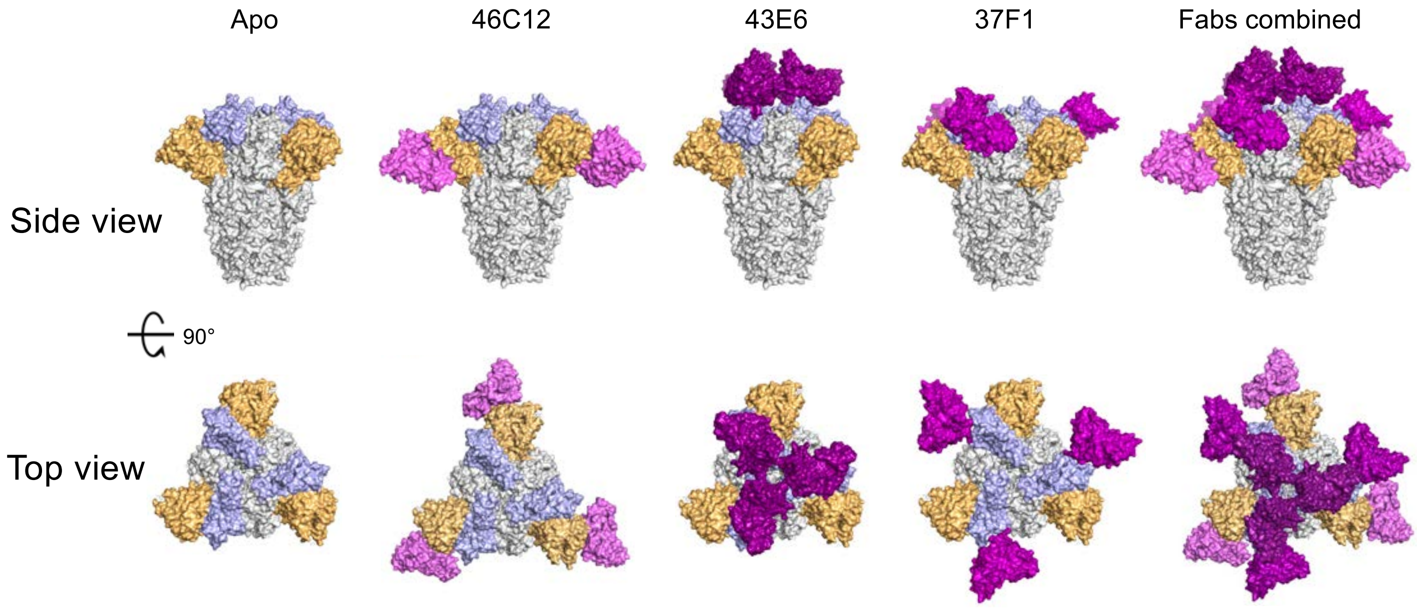

Supplementary Fig. 9 Models of the OC43 S trimer with the bound Fab fragments of 46C12, 43E6 and 37F1, viewed along two orthogonal orientations. Fabs are highlighted in magenta colors, $S1_A$ and the B2 subdomain of $S1_B$ are indicated in light orange and light blue, respectively.

Supplementary Fig. 10

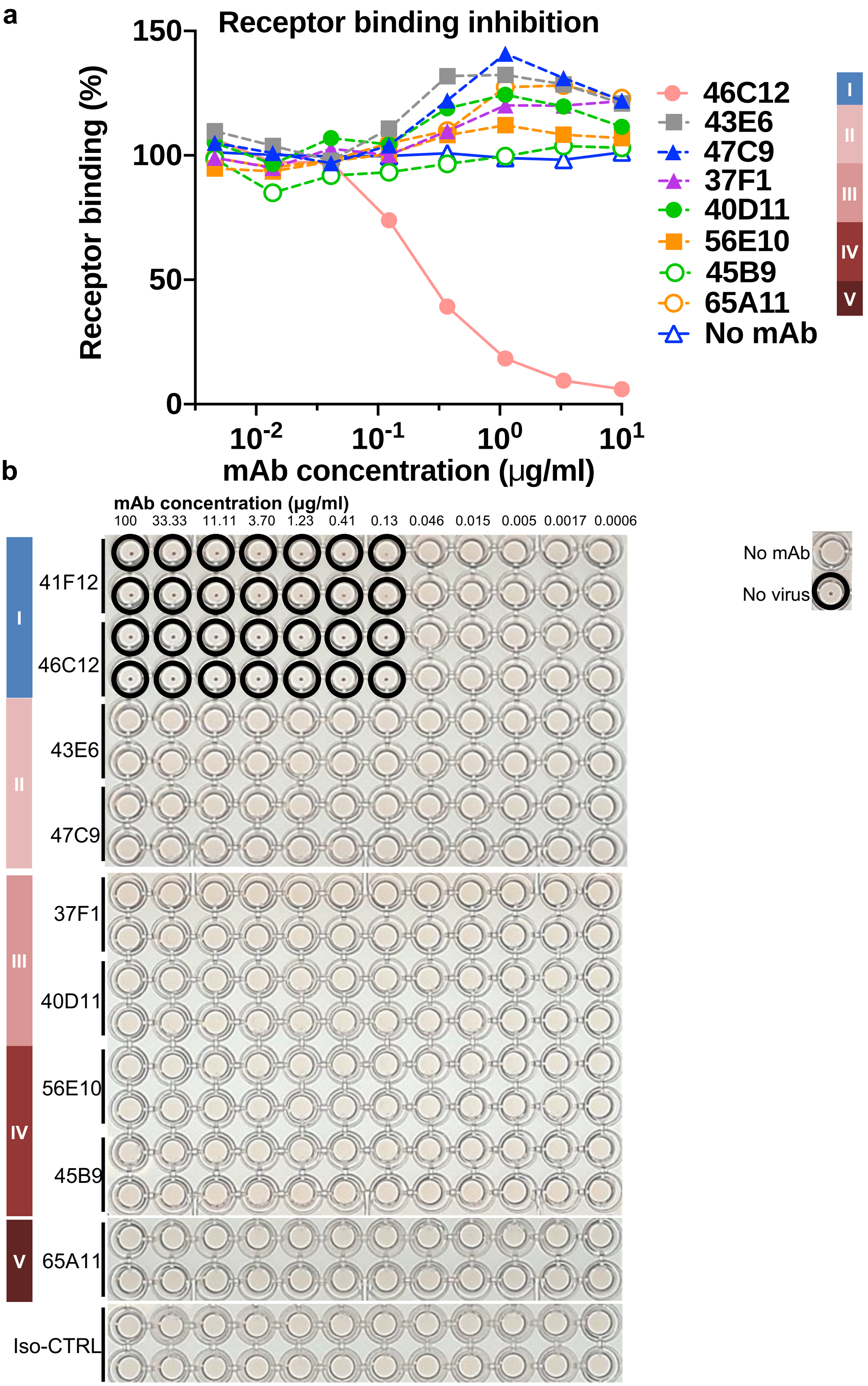

Supplementary Fig. 10 Interference of monoclonal antibodies with sialoglycan-receptor binding. a, Solid-phase Spike-sialoglycan binding inhibition assay: OC43 S_{ecto} pre-incubated with serially diluted OC43 S1-directed mAbs from five antibody competition groups (I-V) was added to ELISA plates coated with bovine submaxillary mucin (BSM) highly enriched in sialoglycan receptors. Binding of OC43 S_{ecto} to BSM was detected using an HRP-conjugated antibody recognizing the C-terminal Strep-tag on OC43 S_{ecto}. Symbols represent mean values from one representative experiment (n=1~4). **b,** Hemagglutination inhibition assay using the OC43 virus to study Interference of monoclonal antibodies with sialoglycan-receptor binding. For the assay, 4 hemagglutinating units of OC43 (strain USA/1967) were mixed with 3-fold serial dilutions of each of the S1A-directed (group I) or S1B-directed (group II-V) monoclonal antibodies in duplicate (starting at 100 μg/ml), or with an isotype control monoclonal antibody (Iso-CTRL). Formation of immune complexes was allowed for 1 h at room temperature prior to incubation with rat erythrocytes at 4˚C in V-shaped 96-well plates. Conditions of erythrocytes incubated without virus (no virus) or monoclonal antibody (no mAb) are shown on the right. Wells showing hemagglutination inhibition are encircled. Two independent experiments were performed, with technical duplicates for each mAb. One representative experiment is shown.

Supplementary Fig. 11

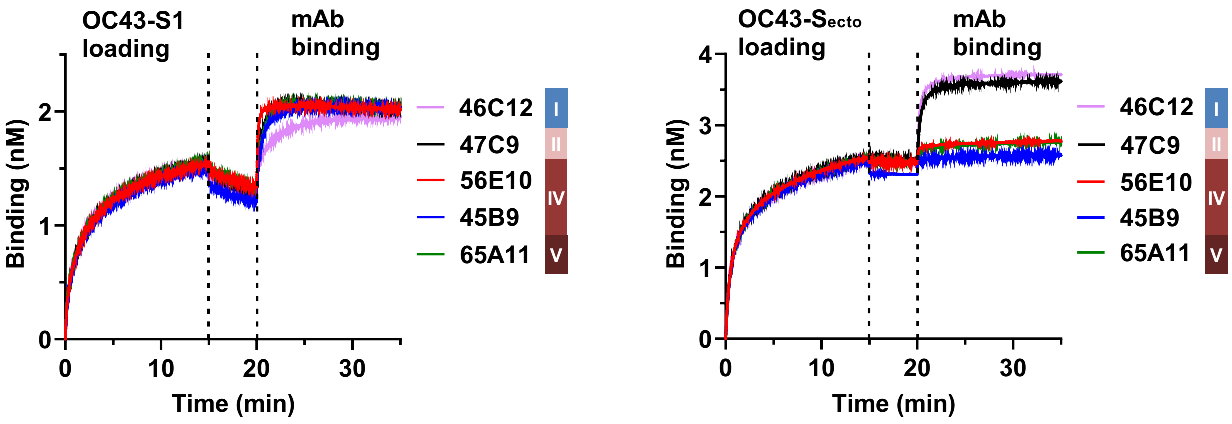

Supplementary Fig. 11 mAb binding to OC43 spike antigens analysed by biolayer interferometry. The OC43-S1 or S_{ecto} was immobilized on the sensor, after a brief washing step, the biosensor tips were immersed into a well containing monoclonal antibody at a concentration of 50 µg/ml for 15 min. 56E10 and 45B9 (both group IV) and 65A11 (group V) mAbs could bind to monomeric OC43 S1, however, no binding to prefusion OC43-S_{ecto} was observed. mAbs 46C12 (group I) and 47C9 (group II) were included as controls. Data from one representative experiment out of two is shown.

Supplementary Fig. 12

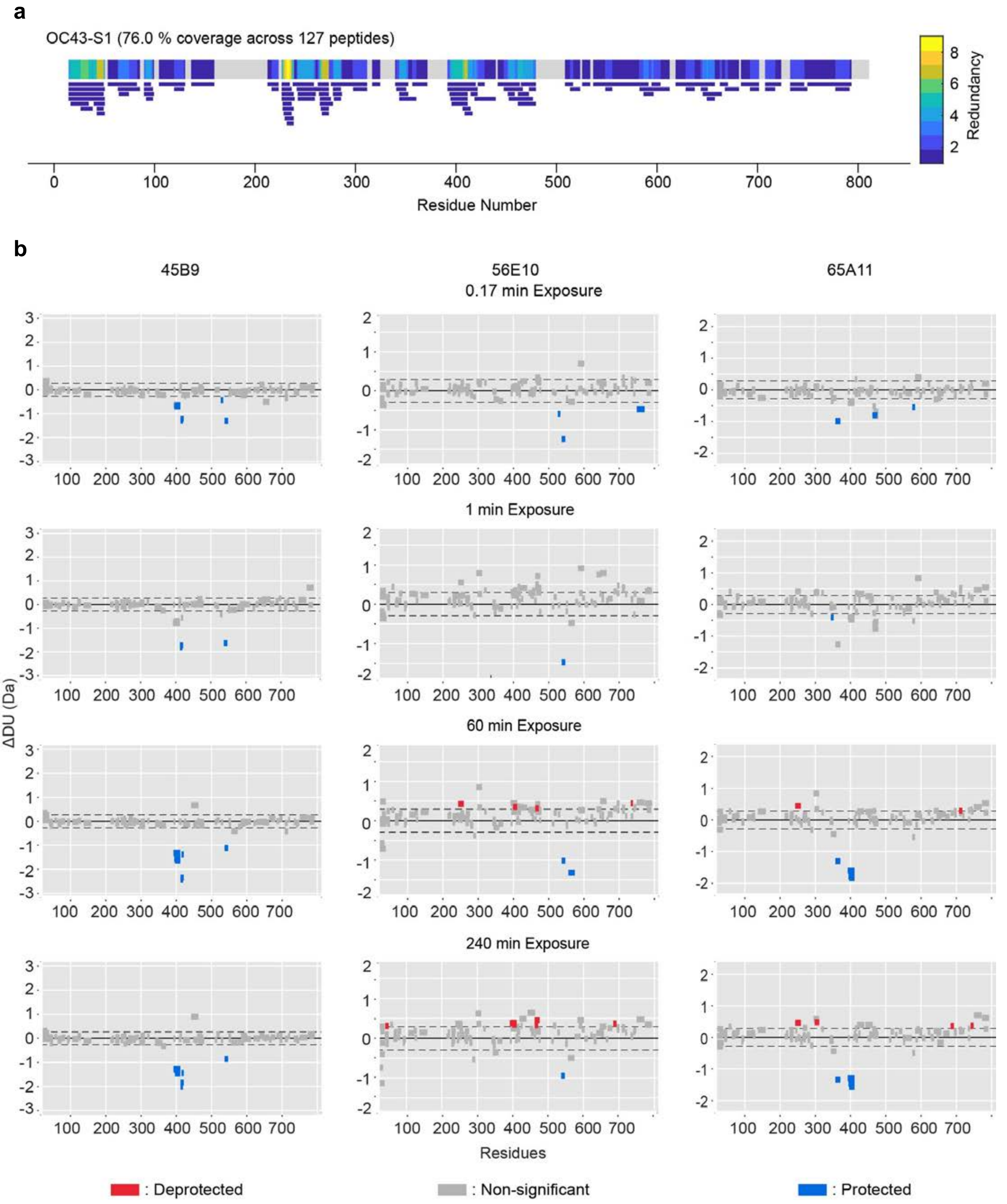

Supplementary Fig. 12 Epitope identification of 56E10, 45B9 and 65A11 Fabs by HDX-MS. a, Coverage map of OC43-S1 sequence depicting accepted peptic peptides and redundancy of these peptides. **b,** Statistical testing for the identification of significant changes in deuterium uptake between the unbound OC43-S1 and Fab-OC43-S1 complex. Hybrid significance test was used for the analysis (Deuteros 2.0). Deuterium uptake difference was evaluated between the two states for each peptide against individual replicate variance and an estimate of measurement error and then tested with two-tailedWelch's t-test to confirm the significance based on individual standard deviations. Significant peptides at different time points are shown in red as deprotected and in blue as protected, 99%, CI: 0.27 Da, p-value < 0.01.

Supplementary Fig. 13

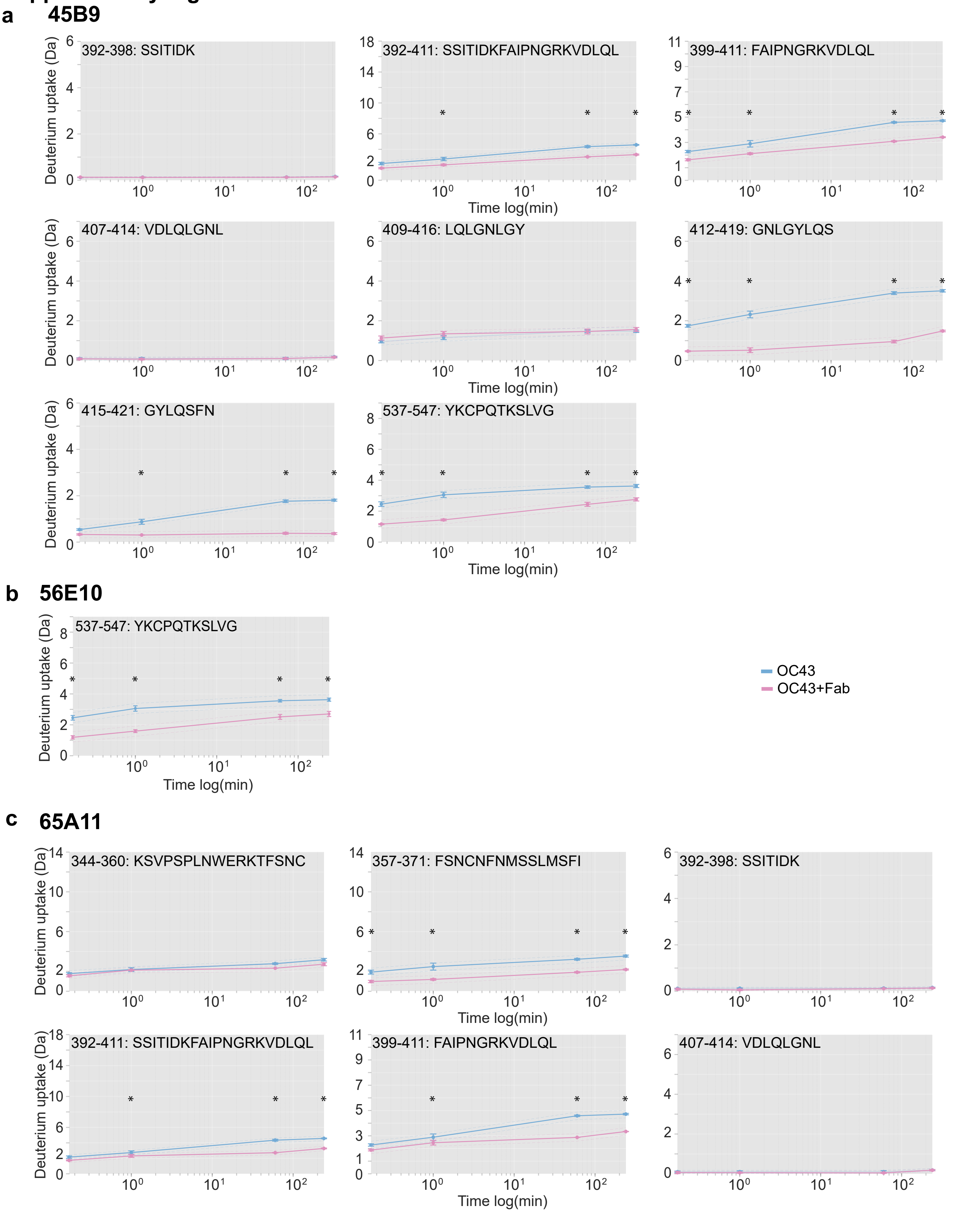

Supplementary Fig. 13 Deuterium uptake plots for the individual peptic peptides employed in epitope mapping of 45B9 (a) 56E10 (b) and 65A11 (c) Fabs by HDX-MS. The HDX-MS measurements were performed on three individual experiments across four time points. Peptide-level significance testing (multiple linear regression, α=0.001, n=3) (Deuteros 2.0) was used for the determination of significant deuterium uptake across the time points for individual peptides between the unbound OC43-S1 (blue) and Fab-OC43-S1 complex (pink). The data is shown as intensity-weighted mean values of deuterium uptake +/- SD for each time point. The significant deuterium uptake per time point is marked with asterisk (p-value < 0.001).

Supplementary Fig. 14

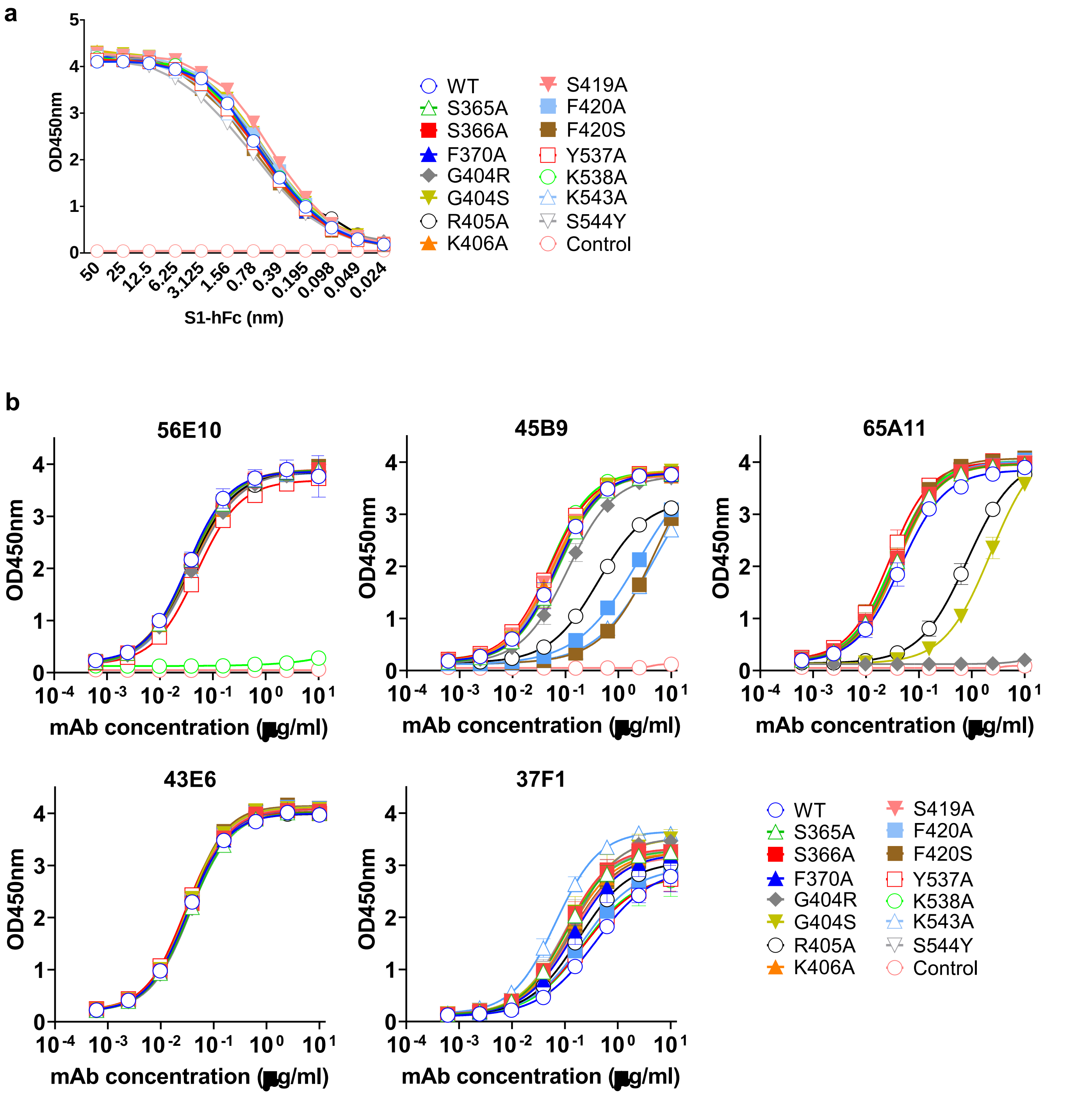

Supplementary Fig. 14 Impact of individual residue substitutions in HDX-MS identified epitope regions on mAb binding. a, OC43 S1 antigens containing individual residue substitutions were expressed from HEK 293T cells and affinity-purified using the C-terminally fused human Fc tag and ELISA plate coating with equimolar amount of S1 antigens was validated using an anti-human Fc antibody. A representative experiment (n=2) is shown. Symbols represent means of duplicates. **b,** ELISA-binding curves of mAbs 56E10, 45B9 and 65A11, and two $S1_B$ control mAbs 43E6 and 37F1 to OC43 S1 antigens containing individual single site mutations. Symbols represent mean values ± SD from two independent experiments with two technical replicates.

Supplementary Fig. 15

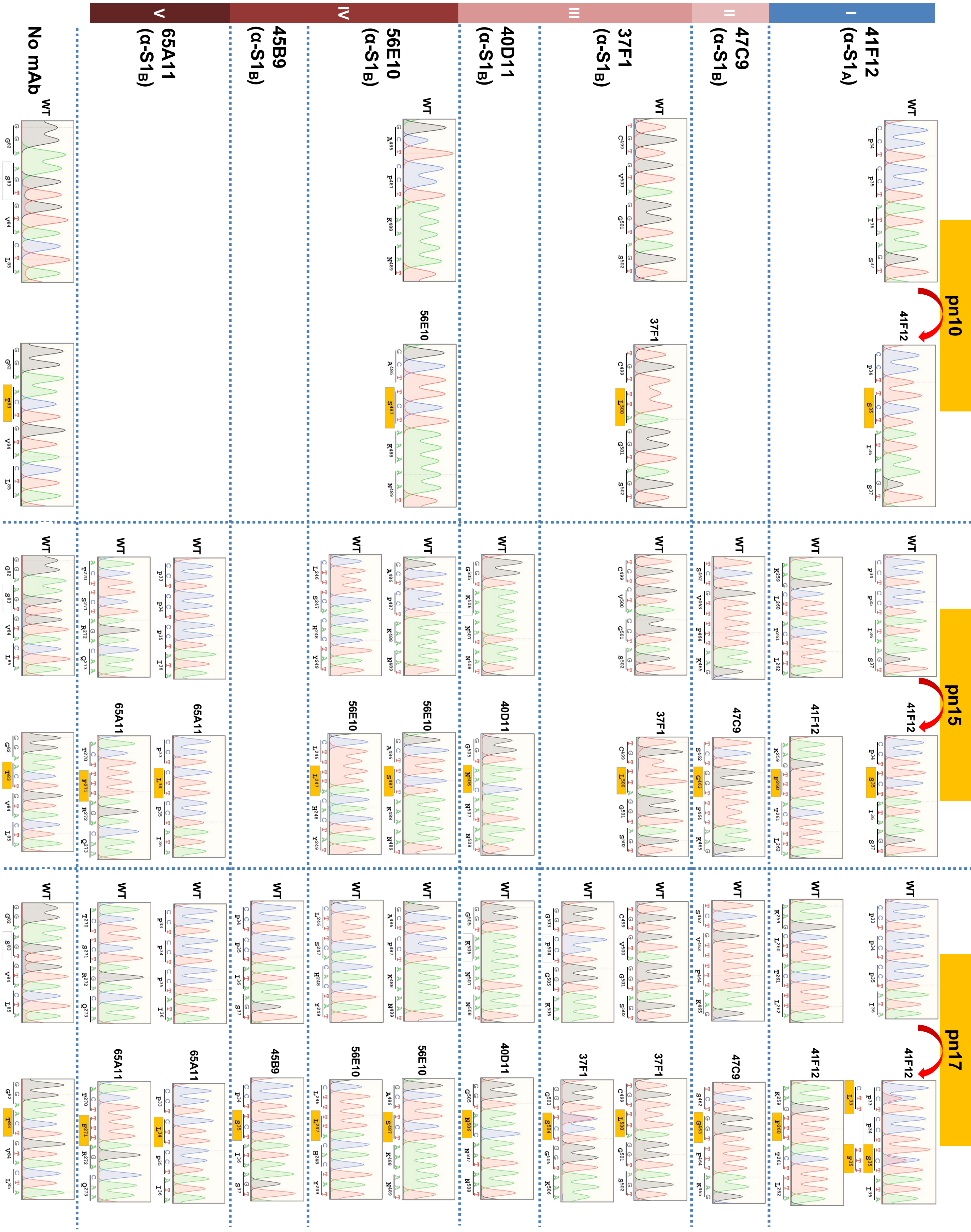

Supplementary Fig. 15 Sequencing of OC43 escape variants selected by neutralizing monoclonal antibodies. Viral RNA from cell culture supernatants from three passages (passage 10, 15 and 17) was isolated using the NucleoSpin RNA Virus kit (Macherey-Nagel), followed by conventional (RT-)PCR and Sanger DNA sequencing of the S gene. Presented are relevant portions of Sanger DNA sequencing chromatograms showing changes at the coding sequence relative to the input virus (W).

Supplementary Fig. 16

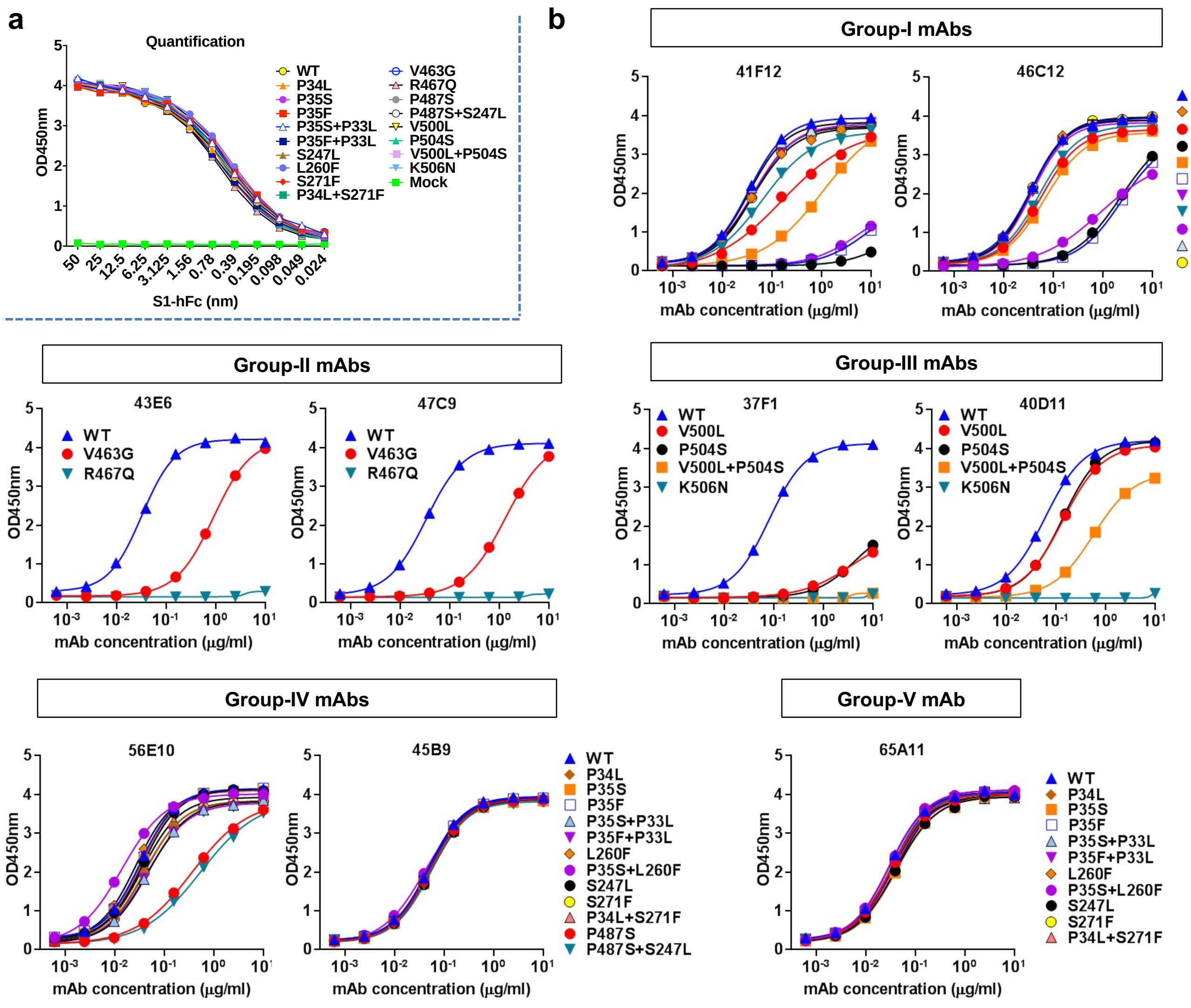

c

Group	mAb	EC$_{50}$ (μg/ml)																		
		WT	P34L	P35S	P35F	P35S+ P33L	P35F+ P33L	S247L	L260F	P35S+ L260F	S271F	P34L+ S271F	V463G	R467Q	P487S	P487S+ S247L	V500L	P504S	V500L+ P504S	K506N
I	41F12	0.034	0.042	0.161	>10	1.048	8.049	0.041	0.064	5.74	0.034	0.042	–	–	–	–	–	–	–	–
	46C12	0.035	0.035	0.057	2.344	0.068	2.369	0.037	0.053	0.895	0.035	0.037	–	–	–	–	–	–	–	–
II	43E6	0.032	–	–	–	–	–	–	–	–	–	–	0.900	>10	–	–	–	–	–	–
	47C9	0.037	–	–	–	–	–	–	–	–	–	–	1.327	>10	–	–	–	–	–	–
III	37F1	0.082	–	–	–	–	–	–	–	–	–	–	–	–	–	–	3.692	4.454	>10	>10
	40D11	0.060	–	–	–	–	–	–	–	–	–	–	–	–	–	–	0.137	0.136	0.575	>10
IV	56E10	0.032	0.039	0.034	0.033	0.047	0.045	0.037	0.027	0.014	0.040	0.036	–	–	0.362	0.540	–	–	–	–
	45B9	0.048	0.050	0.052	0.051	0.051	0.050	0.055	0.048	0.04	0.053	0.048	–	–	0.051	0.053	–	–	–	–
V	65A11	0.030	0.034	0.043	0.034	0.034	0.036	0.036	0.033	0.03	0.041	0.034	–	–	–	–	–	–	–	–

Supplementary Fig. 16 Assessment of selected escape mutations on antibody binding by ELISA. a, OC43 S1 mutants containing escape mutations were expressed from HEK-293T cells and affinity-purified using the C-terminally fused human Fc tag, and equimolar ELISA plate coating of (mutant) S1 antigens was validated using an anti-human Fc antibody. A representative experiment of two independent experiments (n=2) is shown. Symbols represent means of duplicates. **b,** ELISA-binding curves of mAbs to mutant OC43 S1 antigens containing escape mutations. Average values of a representative experiment with two technical replicates is shown. **c,** Half-maximal effective concentration (EC$_{50}$) titers (μg/ml) were determined, based on **b**. S1$_{A}$-reactive mAbs and mutations within S1$_{A}$ domain are colored in blue, and S1$_{B}$-reactive mAbs and mutations within S1$_{B}$ domain are colored in red.

Supplementary Fig. 17

a

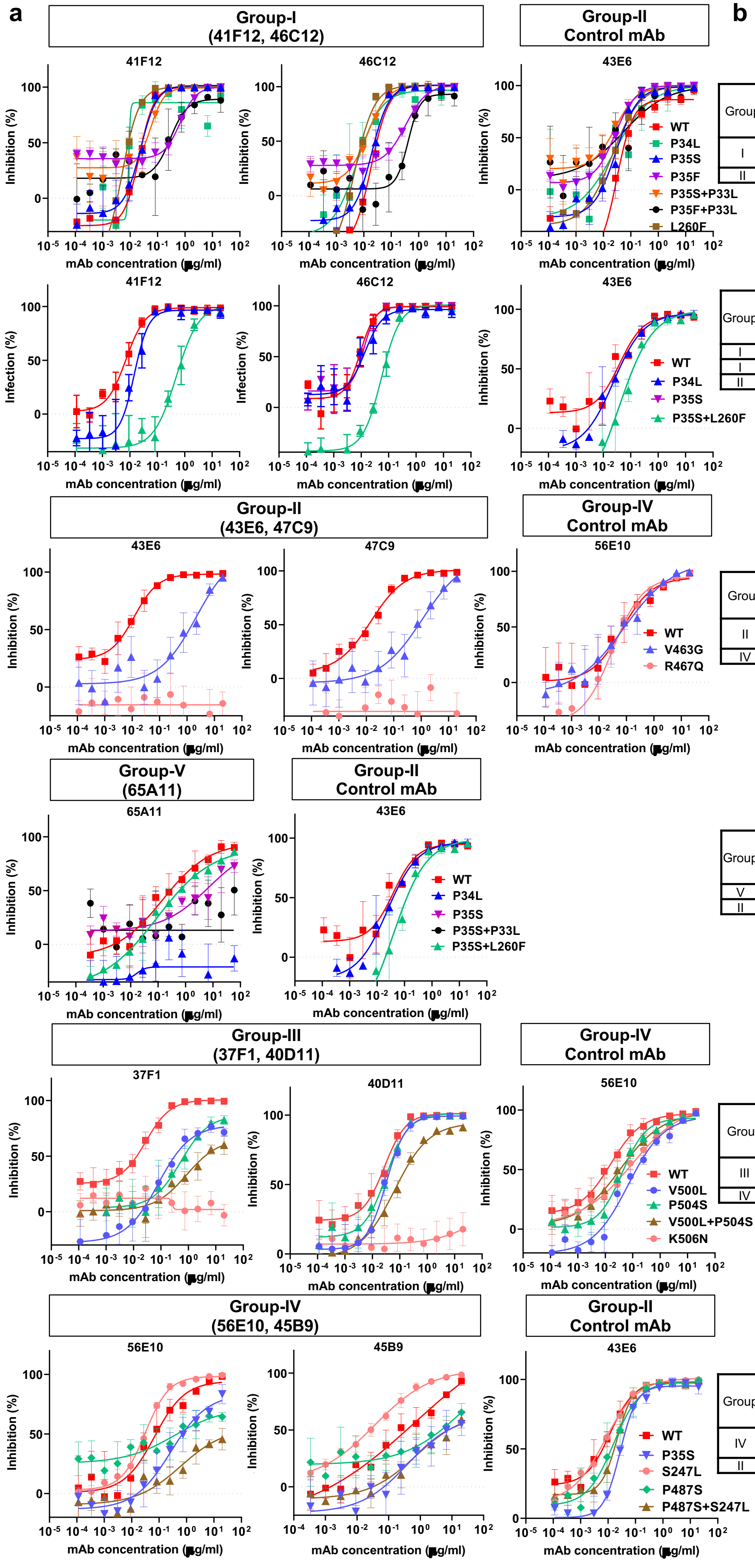

b

Group	mAb	IC$_{50}$ (μg/ml)						
		WT	P34L	P35S	P35F	P35S+P33L	P35F+P33L	L260F
I	41F12	0.021	0.009	0.023	0.665	0.056	0.290	0.005
	46C12	0.019	0.006	0.019	0.334	0.013	0.412	0.005
II	43E6	0.031	0.025	0.029	0.027	0.027	0.052	0.021

Group	mAb	IC$_{50}$ (μg/ml)			
		WT	P34L	P35S	P35S+L260F
I	41F12	0.0072	0.014	–	0.492
I	46C12	0.0092	0.0137	0.0124	0.0546
II	43E6	0.0391	0.0236	–	0.0377

Group	mAb	IC$_{50}$ (μg/ml)		
		WT	V463G	R467Q
II	43E6	0.012	2.14	>20
	47C9	0.015	1.11	>20
IV	56E10	0.068	0.083	0.031

Group	mAb	IC$_{50}$ (μg/ml)				
		WT	P34L	P35S	P35S+P33L	P35S+L260F
V	65A11	0.1465	>60	6.917	>60	0.0897
II	43E6	0.039	0.024	–	–	0.0377

Group	mAb	IC$_{50}$ (μg/ml)				
		WT	V500L	P504S	V500L+P504S	K506N
III	37F1	0.028	0.79	0.53	8.63	>20
	40D11	0.028	0.032	0.032	0.064	>20
IV	56E10	0.016	0.068	0.041	0.035	0.091

Group	mAb	IC$_{50}$ (μg/ml)				
		WT	P35S	S247L	P487S	P487S+S247L
IV	56E10	0.068	0.271	0.031	>20	>20
	45B9	0.667	3.96	0.036	>20	7.45
II	43E6	0.012	0.027	0.0076	0.016	0.022

Supplementary Fig. 17 Assessment of selected escape mutations on antibody neutralization using OC43 S pseudotyped VSV. a, Neutralizing curve of OC43 S mAbs against the viral variants. Single or double amino acid substitutions were introduced to OC43 full-length spike protein to make the pseudotyped virus. Pseudotyped VSV particles pre-incubated with antibodies at indicated concentrations were used to infect HRT-18 cells and luciferase activities in cell lysates were determined at 20 h post transduction to calculate infection inhibition (%) relative to non-antibody-treated control. A representative experiment is shown. Symbols are means $\pm$ SD of n=3~6 replicates. For testing the effect of viral escape mutations on one group of antibodies, an antibody from a non-competing group was used as control. **b,** Neutralizing abilities of mAbs against the viral variants, shown as half-maximal inhibitory concentrations (IC_{50}; μg/ml). $S1_A$-reactive mAbs and mutations within $S1_A$ domain are colored in blue, and $S1_B$-reactive mAbs and mutations within $S1_B$ domain are colored in red likewise.

Supplementary Fig. 18

a

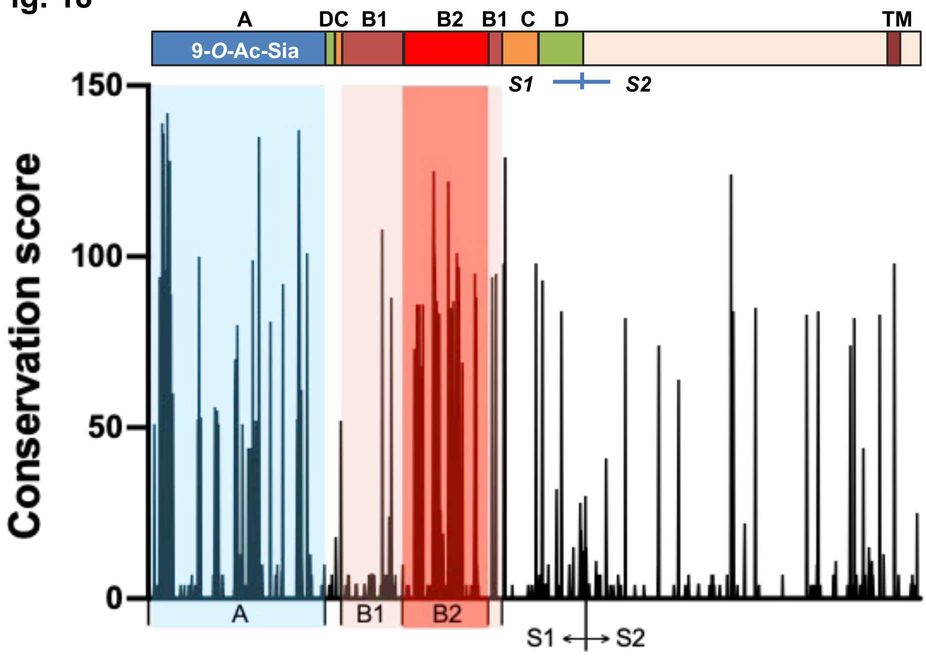

b

GenBank identifiers OC43 spike protein sequences					
AIX10748.1	AGT51620.1	AIV41903.1	AIL49506.1	AIV41819.1	AQN78736.1
AGT51740.1	AGT51511.1	AIV41987.1	AIL49507.1	AIL49519.1	AQN78744.1
AGT51412.1	AGT51491.1	AIL49544.1	AIL49508.1	AIV42011.1	AQN78752.1
AGT51660.1	AGT51730.1	AIL49500.1	AIL49510.1	AIL49505.1	AQN78760.1
AGT51670.1	AIX10750.1	AIV41909.1	AIL49514.1	AIX10760.1	AQN78768.1
AGT51680.1	AIX10749.1	AIL49529.1	AIL49515.1	AOL02453.1	AQN78776.1
AGT51580.1	ATP16757.1	AIV41915.1	AIL49545.1	AMK59677.1	AIX10763.1
AGT51640.1	AIX10752.1	AIL49530.1	AIV41993.1	AIL49509.1	ANZ78838.1
AGT51541.1	AIX10751.1	AIV41921.1	AIL49546.1	AIX09799.1	ANZ78848.1
AGT51471.1	AIX10753.1	AIL49531.1	QRK03842.1	AIX09807.1	ANZ78849.1
AGT51461.1	AIX10754.1	AIV41867.1	AIL49495.1	AIX10761.1	ANZ78834.1
AGT51422.1	AEN19358.1	AIL49532.1	AIL49547.1	AIL49491.1	ANZ78835.1
AGT51690.1	AEN19366.1	AIV41927.1	QRK03832.1	AIV41837.1	ANZ78839.1
AGT51570.1	AIX10755.1	AIL49533.1	AIL49516.1	AIL49492.1	ANZ78840.1
AGT51770.1	AIV41891.1	AIV41933.1	AIL49517.1	AIL49520.1	ANZ78836.1
AGT51720.1	AIL49496.1	AIL49534.1	AIL49549.1	AQN78656.1	ANZ78837.1
AGT51700.1	QHB49097.1	AIV41939.1	AIL49550.1	AIV41849.1	ANZ78841.1
AGT51760.1	AIV41795.1	AIL49535.1	AIL49551.1	AIL49493.1	ANZ78842.1
AGT51481.1	AIL49487.1	AIV41945.1	AIX10759.1	AIV41897.1	ANZ78843.1
AGT51630.1	QHB49098.1	AIL49536.1	AJC98135.1	AIL49494.1	ANZ78844.1
AGT51750.1	AIX10756.1	AIV41951.1	AIL49484.1	AQN78664.1	ANZ78845.1
AGT51600.1	AIV41855.1	AIL49537.1	QRK03852.1	AIX10762.1	ANZ78846.1
AGT51521.1	AIL49497.1	AIV41957.1	AIV41777.1	AQN78672.1	ANZ78847.1
AGT51780.1	AIV41861.1	AIL49538.1	AIL49485.1	AIV41873.1	AVR40342.1
AGT51551.1	AIL49498.1	AIV41963.1	AIL49486.1	AIL49511.1	AVQ05264.1
AGT51710.1	ATP16767.1	AIL49539.1	AIV41783.1	AIV41879.1	AWW13566.1
AGT51402.1	AIL49522.1	AIX10757.1	AIL49488.1	AIL49512.1	AWW13575.1
AGT51650.1	AIL49523.1	AIV41975.1	AIV41969.1	AIV41885.1	AZP73840.1
AGT51431.1	AIL49524.1	AIL49540.1	AIL49501.1	AIL49513.1	AVR40344.1
AGT51561.1	AIL49525.1	AIV41981.1	AJC98127.1	AQN78680.1	QRK03912.1
AGT51790.1	AIL49526.1	AIL49541.1	AIL49489.1	AQN78688.1	QRK03902.1
AGT51451.1	AIL49527.1	AIL49542.1	AIV41831.1	AQN78696.1	QRK03862.1
AGT51590.1	AIL49528.1	AIL49543.1	AIL49502.1	AIV41843.1	QRK03882.1
AGT51441.1	AIL49499.1	AIX10758.1	AIV42005.1	AIL49521.1	QRK03872.1
AGT51800.1	AIV41801.1	AIV41999.1	AIL49503.1	AQN78704.1	QRK03922.1
AGT51531.1	AIV41807.1	AIL49548.1	AIL49518.1	AQN78712.1	QRK03892.1
AGT51610.1	AIV41813.1	AIL49552.1	AIV41789.1	AQN78720.1	QRK03812.1
AGT51501.1	AIV41825.1	AIL49504.1	AIL49490.1	AQN78728.1	QRK03822.1

Supplementary Fig. 18 Sequence conservation of the OC43 S protein. a, S protein sequences derived from 228 different OC43 isolates were retrieved from the NIAID Virus Pathogen Database and Analysis Resource (ViPR). An S protein consensus sequence was generated by majority rule using the MUSCLE multiple sequence alignment tool and polymorphism scores at each residue position are calculated using the VIPR algorithm (https://www.viprbrc.org/) and displayed with the overall topology of the OC43 S protein (as shown in Fig. 1a) as a reference. **b**, OC43 spike protein sequences used to calculate conservation scores.

Supplementary Fig. 19

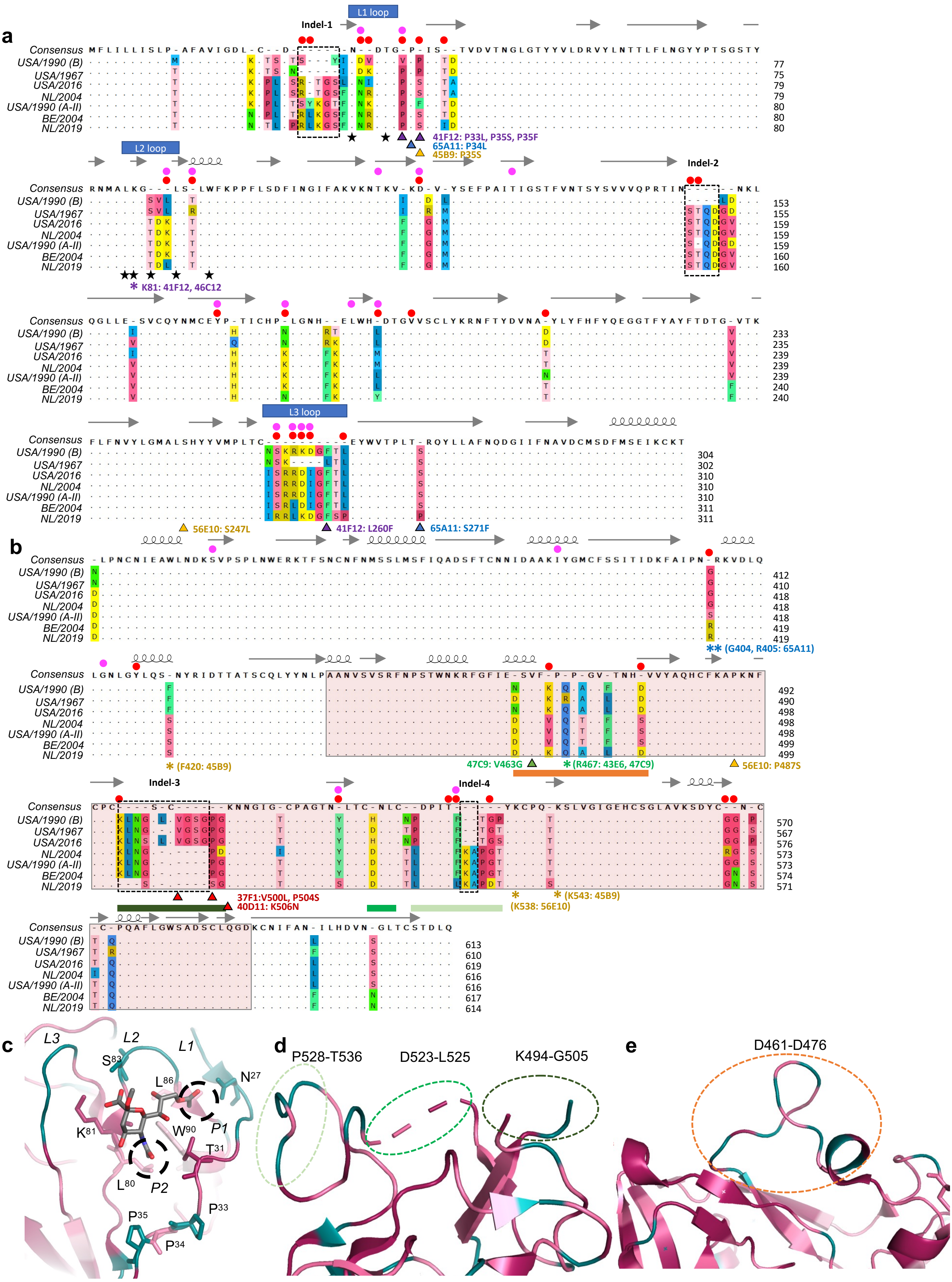

Supplementary Fig. 19 Sequence conservation in OC43 S1 domain. Protein sequence alignment of domain $S1_A$ **(a)** and $S1_B$ **(b)** of S proteins of OC43 strains, using Unipro UGENE ClustalW software. GenBank identifiers of selected S proteins: KF530065.1 (USA/1990(B)), AAT84354.1 (USA/1967), MF314143.1 (USA/2016), OK245433 (NL/2004), KF530088 (USA/1990(A-II)), AY9034551 (BE/2004) and OK245434 (NL/2019). Amino acids that are deviant from the consensus sequence are indicated. Key residues involved in interaction with mAbs identified by escape mutant analysis and mutagenesis are marked with arrow heads and asterisks, respectively. Secondary structural elements (strands and helices) of the HCoV-OC43 prefusion spike (PDB: 6OHW) structure are visualized using ESPript 3.0 (http://espript.ibcp.fr/ESPript/ESPript/). The more variable B2 subdomain of the S1 domain B is outlined by the transparent red box overlay. Sialic acid contacting residues are indicated by star. Loops L1-L3 that constitute the sialic acid binding region are indicated by blue boxes. Residues under positive selection according to Jo et al.[30] are indicated by red dots. Residues under positive selection according to Ren et al.[31] are indicated by purple dots. The four insertions and deletions (Indel-1 to -4) in S1 domains A and B are bordered by dashed rectangles. Three variable loops (K494-G505 loop, D523-L525 loop and P528-T536 loop) in the B2 containing the 37F1 neutralizing epitope are highlighted by green bars, a loop containing region (D461-D476) in B2 that composes a central part of the 47C9 neutralizing epitope is underlined with an orange bar. **c,** Zoomed-in view of the OC43 S1 domain A bound to 9-*O*-acetylated sialic acid (PDB: 6NZK). L1-L3 loops that compose the sialic acid binding site are marked, as well as the P1 and P2 pockets of the receptor binding site. **d and e,** Spike protein conservation in the $S1_{B2}$ subdomain containing the neutralizing epitopes of $S1_B$-directed antibodies. **d,** Zoomed-in view of the tip of the B2 subdomain that comprises the 37F1 epitope, with the three variable and partially unstructured loops encircled. **e,** Zoomed-in view of the B2 subdomain with the loop region (D461-D476) that composes a central part of the 47C9 neutralizing epitope encircled. The encircled loops are shown in colors that correspond to the colored bars in the sequence alignment in **b**. Residues in panels c-e are colored based on their conservation across 228 OC43 S sequences, with the purple to green color gradient represent highly conserved to variable residues.

Supplementary Fig. 20

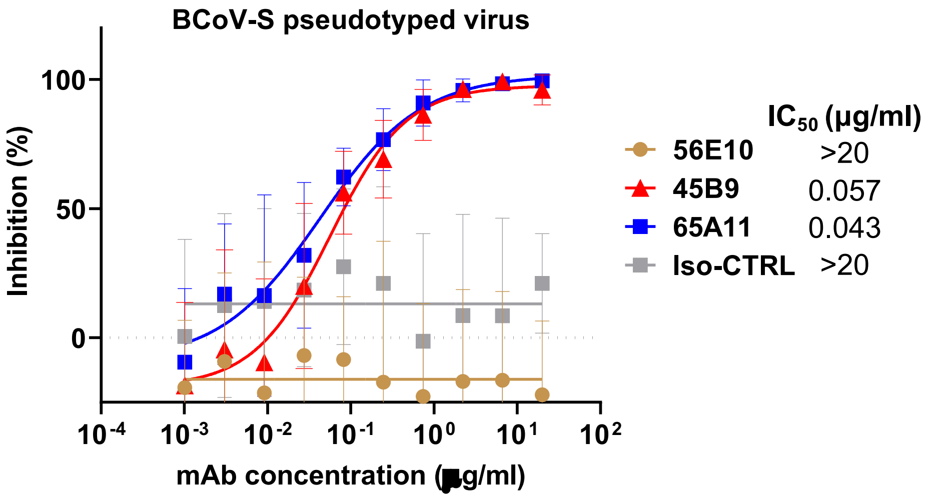

Supplementary Fig. 20 Cross-neutralization with BCoV S pseudovirus. The neutralization capacity of broadly-reactive mAbs 45B9 and 65A11 against BCoV was tested using luciferase-encoding VSV particles pseudotyped with spike protein of BCoV (strain Mebus). Symbols represent mean values ± SD from two independent experiments with six technical replicates are shown**.** mAb 56E10 and an isotype control antibody (Iso-CTRL, an anti-Strep-tag human monoclonal antibody) were used as controls. The IC_{50} values are shown.

Supplementary Fig. 21

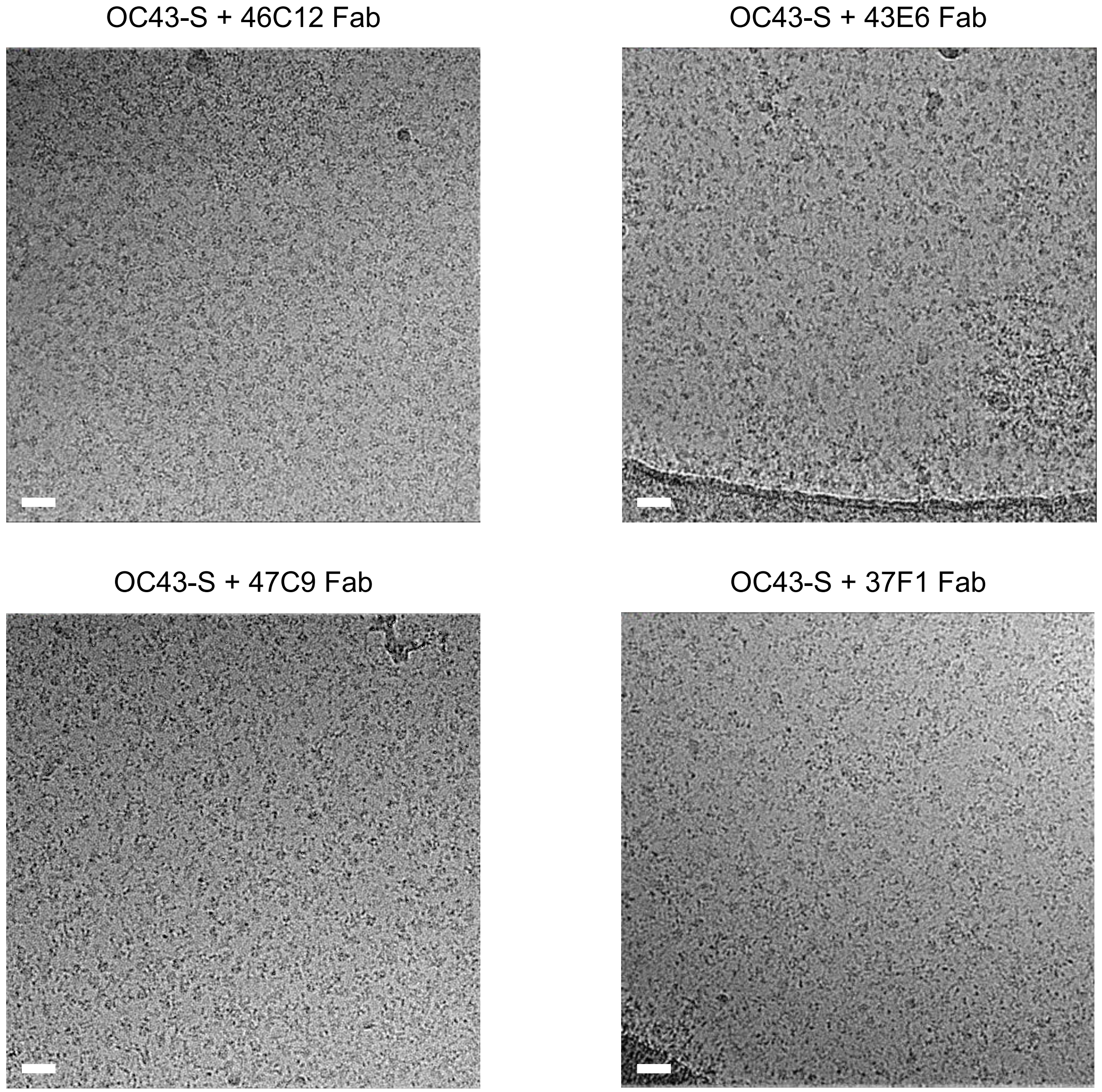

Supplementary Fig. 21 Representative micrographs for OC43 S trimers incubated with the Fab fragments of 46C12, 43E6, 47C9 and 37F1. Scale bar = 30 nm
