# Supplementary material for: Antigenic structure of the human coronavirus OC43 spike reveals exposed and occluded neutralizing epitopes
Source: Nat Commun. 2022 May 25;13:2921. doi: 10.1038/s41467-022-30658-0 (PMC9132891; doi:10.1038/s41467-022-30658-0)
Supplement: Supplementary file 2 — Reporting Summary [file 41467_2022_30658_MOESM2_ESM.pdf]

nature portfolio

Corresponding author(s): Berend-Jan Bosch

Last updated by author(s): April 15, 2022

Reporting Summary

Nature Portfolio wishes to improve the reproducibility of the work that we publish. This form provides structure for consistency and transparency in reporting. For further information on Nature Portfolio policies, see our Editorial Policies and the Editorial Policy Checklist.

Statistics

For all statistical analyses, confirm that the following items are present in the figure legend, table legend, main text, or Methods section.

n/a	Confirmed	
☐	☒	The exact sample size (n) for each experimental group/condition, given as a discrete number and unit of measurement
☐	☒	A statement on whether measurements were taken from distinct samples or whether the same sample was measured repeatedly
☐	☒	The statistical test(s) used AND whether they are one- or two-sided<br>*Only common tests should be described solely by name; describe more complex techniques in the Methods section.*
☒	☐	A description of all covariates tested
☒	☐	A description of any assumptions or corrections, such as tests of normality and adjustment for multiple comparisons
☐	☒	A full description of the statistical parameters including central tendency (e.g. means) or other basic estimates (e.g. regression coefficient) AND variation (e.g. standard deviation) or associated estimates of uncertainty (e.g. confidence intervals)
☐	☒	For null hypothesis testing, the test statistic (e.g. F, t, r) with confidence intervals, effect sizes, degrees of freedom and P value noted<br>*Give P values as exact values whenever suitable.*
☒	☐	For Bayesian analysis, information on the choice of priors and Markov chain Monte Carlo settings
☒	☐	For hierarchical and complex designs, identification of the appropriate level for tests and full reporting of outcomes
☒	☐	Estimates of effect sizes (e.g. Cohen's d, Pearson's r), indicating how they were calculated

Our web collection on statistics for biologists contains articles on many of the points above.

Software and code

Policy information about availability of computer code

Data collection	ELISA plate reader (EL-808, Biotek) was used to collect ELISA data. EVOS FL fluorescence microscope (Thermo Fisher Scientific, the Netherlands) was used to collect fluorescence images. The Octet (Octet RED384, Fortebio) was used to collect affinity data and epitope binning data. Berthold Centro LB 960 plate luminometer was used to collect firefly luciferase data. Amersham Typhoon Biomolecular Imager (channel Cy2; resolution 10mm; GE Healthcare) was used to collect fluorescence images. Titan Krios (ThermoFisher Scientific) was used to collect cryo-EM data. Separated peptides were analyzed with XEVO G2 mass spectrometer (Waters Corp., USA). Full length OC43 S genes deposited until July 2021 were retrieved from GenBank. Online protein sequence alignment tool: http://www/ebi/ac/uk/Tooll/msa/clustalo/.
Data analysis	GraphPad Prism (version 8), Fortebio Data Analysis 7.0, ImageQuantTL 8.2, Relion version 3.0.1 and 3.1.1, MOTIONCOR2, CTFFIND-4.1, UCSF Chimera 1.15.0, Phyre2 server, DeepEMhancer 0.13, COSMIC2, Molprobity 4.02-528, EMringer 1.0.0, SBGrid, PDBePISA 1.52, LigPlot+ 2.2, PLGS 3.0.1, DynamX 3.0, Deuteros 2.0, MEGA X, Phenix 1.19.2_4158, UCSF ChimeraX 1.2.5, Waters PLGS 3.0.1, Waters DynamX 3.0

For manuscripts utilizing custom algorithms or software that are central to the research but not yet described in published literature, software must be made available to editors and reviewers. We strongly encourage code deposition in a community repository (e.g. GitHub). See the Nature Portfolio guidelines for submitting code & software for further information.

Data

Policy information about availability of data

All manuscripts must include a data availability statement. This statement should provide the following information, where applicable:

- Accession codes, unique identifiers, or web links for publicly available datasets
- A description of any restrictions on data availability
- For clinical datasets or third party data, please ensure that the statement adheres to our policy

Data underlying Figs. 1c, 2a–b, 3d, 3e, 5c, 6b–c, 7c-d, Supplementary Figs 1d-e, 5, 10, 14, 16, 17 and 20 are provided as Source Data files in a publicly accessible repository (https://figshare.com/s/84afe2bace94f17d73ef). PDB files of OC43 spike protein (PDB ID: 6NZK and 6OHW) were downloaded from NCBI database (https://www.ncbi.nlm.nih.gov/). Spike protein sequences used in this study were downloaded from NCBI database (https://www.ncbi.nlm.nih.gov/) (See supplementary table 2 for the accession numbers). Sequences of the monoclonal antibodies characterized here are available from GenBank under the following accession numbers: OK245435 [https://www.ncbi.nlm.nih.gov/nuccore/OK245435], OK245436 [https://www.ncbi.nlm.nih.gov/nuccore/OK245436], OK245437 [https://www.ncbi.nlm.nih.gov/nuccore/OK245437], OK245438 [https://www.ncbi.nlm.nih.gov/nuccore/OK245438], OK245439 [https://www.ncbi.nlm.nih.gov/nuccore/OK245439], OK245440 [https://www.ncbi.nlm.nih.gov/nuccore/OK245440], OK245441 [https://www.ncbi.nlm.nih.gov/nuccore/OK245441], OK245442 [https://www.ncbi.nlm.nih.gov/nuccore/OK245442], OK245443 [https://www.ncbi.nlm.nih.gov/nuccore/OK245443], OK245444 [https://www.ncbi.nlm.nih.gov/nuccore/OK245444], OK245445 [https://www.ncbi.nlm.nih.gov/nuccore/OK245445], OK245446 [https://www.ncbi.nlm.nih.gov/nuccore/OK245446], OK245447 [https://www.ncbi.nlm.nih.gov/nuccore/OK245447], OK245448 [https://www.ncbi.nlm.nih.gov/nuccore/OK245448], OK245449 [https://www.ncbi.nlm.nih.gov/nuccore/OK245449], OK245450 [https://www.ncbi.nlm.nih.gov/nuccore/OK245450], OK245451 [https://www.ncbi.nlm.nih.gov/nuccore/OK245451] and OK245452 [https://www.ncbi.nlm.nih.gov/nuccore/OK245452]. The accession numbers for the cryo-EM structures of HCoV-OC43 S with 46C12/43E6/47C9 Fab reported in this paper is PDB ID 7PNM [https://www.ncbi.nlm.nih.gov/structure/?term=7PNM], 7PNQ [https://www.ncbi.nlm.nih.gov/structure/?term=7PNQ] and 7PO5 [https://www.ncbi.nlm.nih.gov/structure/?term=7PO5] respectively. Source data are provided with this paper.

Field-specific reporting

Please select the one below that is the best fit for your research. If you are not sure, read the appropriate sections before making your selection.

- [x] Life sciences
- [ ] Behavioural & social sciences
- [ ] Ecological, evolutionary & environmental sciences

For a reference copy of the document with all sections, see nature.com/documents/nr-reporting-summary-flat.pdf

Life sciences study design

All studies must disclose on these points even when the disclosure is negative.

Sample size	For infection experiments, 50,000 cells were taken per sample. Infection dose of authentic virus infection experiments was based on MOI to prevent double infections of cells, or on luciferase counts (for luciferase-based VSV pseudotyping exp.) to be in linear range of measurement. The sample size was sufficient for a good statistical analysis based on previous experience (PMID: 32366817).
Data exclusions	No data were excluded from the analyses.
Replication	Experimental findings were reliably reproduced. Most of the experiments were replicated two or three times, as noted in the figure legends and text.
Randomization	All samples were allocated in random order, no particular bias is envisaged.
Blinding	Blinding was not relevant as the reported data was not based on subjective observations, but quantitative measurements, including ELISA etc.

Reporting for specific materials, systems and methods

We require information from authors about some types of materials, experimental systems and methods used in many studies. Here, indicate whether each material, system or method listed is relevant to your study. If you are not sure if a list item applies to your research, read the appropriate section before selecting a response.

Materials & experimental systems

n/a	Involved in the study	
[ ]	[x]	Antibodies
[ ]	[x]	Eukaryotic cell lines
[x]	[ ]	Palaeontology and archaeology
[x]	[ ]	Animals and other organisms
[x]	[ ]	Human research participants
[x]	[ ]	Clinical data
[x]	[ ]	Dual use research of concern

Methods

n/a	Involved in the study	
[x]	[ ]	ChIP-seq
[x]	[ ]	Flow cytometry
[x]	[ ]	MRI-based neuroimaging

Antibodies

Antibodies used	1. StrepMAB-Classic, HRP conjugate, Cat.no: 2-1509-001, IBA, 1:2,000<br>2. Goat anti-Human IgG (H+L) Cross-Adsorbed Secondary Antibody Alexa Fluor 488, Catalog # A-11013, Invitrogen, 1:200<br>3. Goat anti-human IgG (HRP), Catalog #2040-05, ITK Southern Biotech, 1:2000<br>4. Mouse anti-rat IgG1/2b/2c, cat. no. KT96/KT98/KT99, Absea Biotechnology Ltd., 1:2000<br>5. Anti-streptag monoclonal antibody (PMID: 30938227)<br>6. OC43 S specific antibodies isolated from humanized H2L2 mice (PMID: 33731724)
Validation	Validation of the OC43 S specific antibodies was done by ELISA binding assays, antibody binding competition assays and virus neutralization assays, as detailed in this study. Validation of anti-streptag isotype control antibody was described earlier (PMID: 30938227). All the secondary antibodies were bought from commercial vendors and were validated by the manufacturers.

Eukaryotic cell lines

Policy information about cell lines

Cell line source(s)	Human embryonic kidney cell, HEK293T, from ATCC® CRL-11268<br>Human embryonic kidney cell, HEK293S GnTI-, from ATCC® CRL-3022™<br>Human colon cell, HRT-18, from ATCC® CCL-244™
Authentication	The cell lines were not authenticated further after purchase.
Mycoplasma contamination	We confirm that all cells were tested as mycoplasma negative.
Commonly misidentified lines<br>(See ICLAC register)	No commonly misidentified cell lines were used.
